# Data-driven model reveals increased stability of CAG-expanded *huntingtin* RNA due to MID1 binding

**DOI:** 10.1371/journal.pcbi.1014342

**Published:** 2026-06-02

**Authors:** Yuhong Liu, Annika Reisbitzer, Domagoj Dorešić, Jan Hasenauer, Sybille Krauß, Tatjana Tchumatchenko

**Affiliations:** 1 Life and Medical Sciences (LIMES) Institute, University of Bonn, Bonn, Germany; 2 Bonn Center for Mathematical Life Sciences, University of Bonn, Bonn, Germany; 3 Institute of Biology, University of Siegen, Siegen, Germany; 4 Institute of Experimental Epileptology and Cognition Research, University of Bonn Medical Center, Bonn, Germany; Clemson University, UNITED STATES OF AMERICA

## Abstract

RNA-binding proteins (RBP) are important regulators of RNA metabolism. In neurodegenerative disorders such as Huntington’s Disease (HD), disrupted RBP-RNA interactions contribute to neuronal dysfunction. One such RBP, Midline 1 (MID1), has been shown to aberrantly associate with mutant huntingtin (*Htt*) RNA, enhancing its translation, yet the mechanism driving this effect remains unknown. Here, we develop a computational model to understand the role of MID1. Based on previously published data, our model predicts that MID1 increases the stability of the *Htt* RNA. We experimentally validate this prediction, showing that overexpression of MID1 significantly prolongs the half-life of mutant *Htt* RNA. Furthermore, we evaluate model refinements, including clustering of MID1-bound RNA, which allow capturing all key observations in the data. Together, we provide a data-driven framework that underlines the importance of RBP-RNA interaction in post-transcriptional regulation. This framework also shows how individual molecular reactions jointly determine RNA stability and protein levels in HD.

## Author summary

Huntington’s Disease is a severe brain disorder caused by a mutation that makes a part of the huntingtin gene much longer than normal. This change affects how the gene’s RNA interacts with proteins that normally control how long RNAs live and how much protein they produce. One of these proteins, called MID1, attaches more strongly to the mutant huntingtin RNA and increases the amount of mutant huntingtin protein, which may worsen the disease.

In this study, we use mathematical modeling together with experimental data to understand how MID1 affects huntingtin RNA. Our model suggests that MID1 makes the RNA more stable, allowing it to live in the cell longer and produce more protein. We confirm this prediction in biological experiments. We also find that RNAs bound to MID1 may group together inside cells, further boosting protein production. Our findings show how combining modeling and experiments can uncover hidden mechanisms in diseases like Huntington’s Disease and may help identify new ways to control harmful protein buildup inside cells.

## Introduction

Translation of RNA into protein is a fundamental cellular process that influences the amount of proteins available for cellular activity [1,2]. Such a process is subject to multilayered regulation, both temporally [3] and spatially [4–6], and even minor perturbations can result in severe cellular dysfunction [7–9]. A central mechanism for this regulation involves interactions between RNA and RNA-binding proteins (RBP), which coordinate key steps of RNA metabolism such as splicing, localization, stability, and translation efficiency [10–12]. Disruptions in these interactions are particularly detrimental in neurons, where RNA processing plays a pivotal role in maintaining neuronal integrity and function [13,14]. Indeed, aberrant RBP activity is increasingly recognized as a pathogenic driver in various neurological disorders [15,16].

**Fig 1:**
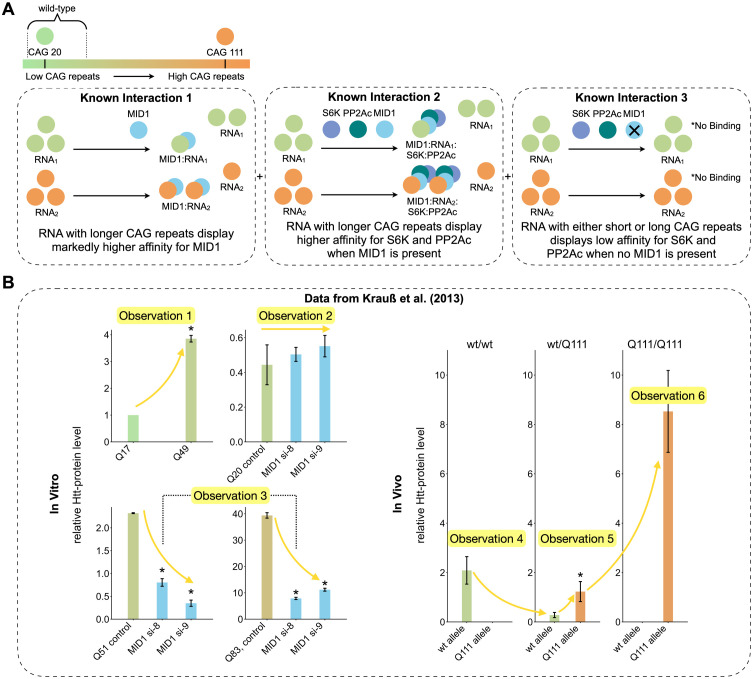
Visual summary of impact of CAG-expansion, and the experimental data used for parameter inference. **(A)** Schematic representation of MID1-dependent RNP complex formation. The legend indicates the color scheme used to represent different CAG repeat or glutamine (Q) lengths. Wild-type is defined as when the CAG repeat or glutamine length is less than 36. **(B)** Experimental *in vitro* and *in vivo* data taken from [20]. In the *in vitro* panels, the x-axis labels, e.g., Q17, indicate constructs with the corresponding number of CAG repeats, while in the *in vivo* panels, the subtitles, e.g., wt/wt, denote mouse genotypes. MID1 si-8 and MID1 si-9 refer to two independent small interfering RNA sequences specifically designed to knock down MID1 expression. The error bars represent the standard deviation of the data. An asterisk above a bar indicates a significant difference (*p* < 0.05) relative to the first bar in the subplot. All *in vivo* data originate from the same western blot experiment and are directly comparable across genotypes. Bars are absent in the wt/wt and Q111/Q111 subplots where the respective allele is not expressed.

A prominent example is Huntington’s Disease (HD), a neurodegenerative disorder caused by an expansion of the CAG trinucleotide repeats in the huntingtin gene (*Htt*) [17,18]. The CAG trinucleotide encodes glutamine (Q) in the huntingtin protein (HTT), so, in the following, we use Q number and CAG repeat length interchangeably. The number of CAG repeats in *Htt* RNA is typically fewer than 36 in the wild-type (wt) [19]. Mutant *Htt* RNA, however, contains longer CAG repeats, which fold into stable hairpin structures [20, 21]. These aberrant secondary structures found in *Htt* RNA with expanded CAG repeat lengths are preferentially recognized by the RBP Midline 1 (MID1) (Fig 1A, known interaction 1), an E3 ubiquitin ligase tagging proteins for degradation [22,23]. This specific interaction between MID1 and mutant *Htt* RNA can thus potentially modify RNA metabolism.

Upon binding to *Htt* RNA, MID1 orchestrates the assembly of a translationally active ribonucleoprotein (RNP) complex that includes protein phosphatase 2A (PP2A) and S6 kinase (S6K) [21,24]. This interaction also occurs in a CAG repeat length dependent manner [20]: RNA with longer CAG repeat length (e.g., 111 CAG repeats) displays markedly higher affinity for MID1 and its associated complex compared to those with physiological repeat lengths (e.g., 20 CAG repeats) (Fig 1A, known interaction 2). The binding affinity is length-dependent because the CAG-containing RNA hairpin progressively elongates and stabilizes as the CAG repeat length increases, thereby providing a larger binding platform for MID1 [20]. Notably, knockdown of MID1 abolishes PP2A and S6K association with *Htt* RNA, establishing MID1 as a necessary scaffold for complex formation (Fig 1A, known interaction 3) [20].

These recruited complex compounds, PP2A and S6K, modulate phosphorylation states of translation regulators, thereby enhancing translation initiation of the mutant RNA. The downstream consequence of these interactions is an increase in translation efficiency from RNA with expanded repeats. This is evident in published data for *in vitro* and *in vivo* experiments (Fig 1B) [20]. In *in vitro* experiments, translation of *Htt* RNA constructs bearing high CAG repeat length yields substantially more protein (e.g., Q49, which indicates HTT with a polyglutamine stretch of 49 residues) than constructs with lower CAG repeat length (e.g., Q17), despite similar RNA levels. This effect is abolished upon MID1 knockdown using MID1-specific short interfering RNA (MID1 si-8 or si-9) for constructs with expanded repeats, while having minimal or no effect on low-repeat constructs—likely due to the weak binding affinity between MID1 and wt *Htt* RNA [20].

Furthermore, additional *in vivo* evidence from knock-in mouse models carrying the mutant *Htt* allele (Q111/+) suggests that simple binding interactions might not be sufficient to explain the observed data (Fig 1B *in vivo* panel). In both heterozygous and homozygous mutant mice, the mutant allele consistently produces higher levels of HTT than the wt allele, mirroring the *in vitro* findings. However, the data also reveals more complex protein level patterns that cannot be explained by a simple linear increase in MID1 binding. For example, in heterozygous mice, while the mutant allele shows significantly higher protein output than the wt allele as expected, the total amount of protein is comparable with that in homozygous wt mice. This suggests that MID1 binding alone does not fully account for the differences. Moreover, although homozygous mice carry twice as many mutant alleles as heterozygous mice, their HTT levels are more than double, indicating a supralinear increase that cannot be explained by allele dosage alone.

These experimental findings together demonstrate that MID1 increases the translation of RNA with expanded CAG repeats. While the role of MID1 in recruiting translation-associated kinases is well established, the quantitative link between these molecular interactions and the resulting differences in protein expression remains unresolved. In particular, it is still unclear how the difference in CAG repeat length, MID1 binding affinity, and co-factor recruitment jointly determine translation efficiency and drive the observed expression patterns. Furthermore, these observations indicate that other mechanisms introducing nonlinear dependencies, such as competitive or cooperative effects, may be at play beyond the simple binding affinity or allele dosage.

Mathematical modeling is a powerful approach to unravel such mechanisms. Ordinary differential equation (ODE) models have already proven to be powerful tools for elucidating the quantitative dynamics of RNA–protein interactions [25,26], as well as autoregulatory feedback loops of RBP [27]. Furthermore, ODE models have been used to investigate the role of the yeast RBP Puf3 in regulating RNA stability [28]. However, despite these advances, such quantitative frameworks have yet to be extensively applied to understand RBP-driven RNA translation dynamics in neurodegenerative diseases like HD.

Our work addresses this gap by developing an ODE model describing the influence of CAG repeat length on MID1 binding and the resulting HTT levels based on the data [20] described above (Fig 1B). We derive testable predictions, such as the impact of MID1 binding on *Htt* RNA stability. To evaluate these predictions, we conduct biological experiments. Furthermore, we use parameter estimation and model selection to assess which model best describes the available data. By integrating mathematical modeling with experimental data, our findings provide a new mechanistic understanding of how MID1 influences RNA regulation and offer potential targets for therapeutic intervention in disorders characterized by abnormal RNA stability and translation dynamics.

## Results

### MID1-dependent core molecular interactions alone do not account for the observed protein level patterns

To systematically uncover how MID1 influences HTT level, we consider previously published experimental data regarding several characteristic observations of HTT level in the presence of expanded CAG repeats (Fig 1B) [20]. These observations are:

(Observation 1) HTT levels increase with the CAG repeat length.
(Observation 2) MID1 level has a minimal effect at low CAG repeat lengths.
(Observation 3) MID1 level modulates HTT levels at high CAG repeat lengths.
(Observation 4) The wt allele produces a lower HTT level in wt/Q111 compared to wt/wt mice.
(Observation 5) In wt/Q111 mice, the Q111 allele is preferentially translated.
(Observation 6) Q111/Q111 mice show a supralinear increase in the HTT level from the Q111 allele in wt/Q111 mice.

To evaluate whether these characteristics can be explained solely by the core molecular interactions between *Htt* RNA, MID1, and downstream effectors, we develop a *Baseline Model*. This model captures the previously identified core molecular interactions (Fig 1A), including allele-specific MID1 binding, MID1-dependent recruitment of the S6K, phosphorylation-dependent translation, and protein degradation.

The *Baseline Model* considers two *Htt* RNAs (one from each allele; denoted RNA_1_, RNA_2_), unbound MID1, MID1-bound RNA complexes (MID1:RNA_1_, MID1:RNA_2_), unphosphorylated and phosphorylated S6K (S6K, S6K^P^), and the resulting HTT (HTT_1_, HTT_2_). The concentrations of all biochemical species in the model are encoded in the state vector:


$$\begin{array}{c} x=([{RNA}_{1}],[{RNA}_{2}],[MID1],[{MID1:RNA}_{1}], \end{array}$$

$$[{MID1:RNA}_{2}],[S6K],[{S6K}^{P}],[{\mathrm{HTT}}_{1}],[{\mathrm{HTT}}_{2}]{)}^{⊤},$$

The characteristics of RNA_1_ and RNA_2_, i.e., CAG repeat lengths *q* of each allele, *q*_1_ and *q*_2_, depend on the specific experimental context. For instance, for an experiment with heterozygous wt/Q111 mice, the RNA_1_ is the wt RNA and RNA_2_ is the Q111 RNA. As the model structure is symmetric with respect to RNA_1_ and RNA_2_, the order of the assignment will not influence results (see Sec. 1.1 in S1 Appendix).

**Fig 2:**
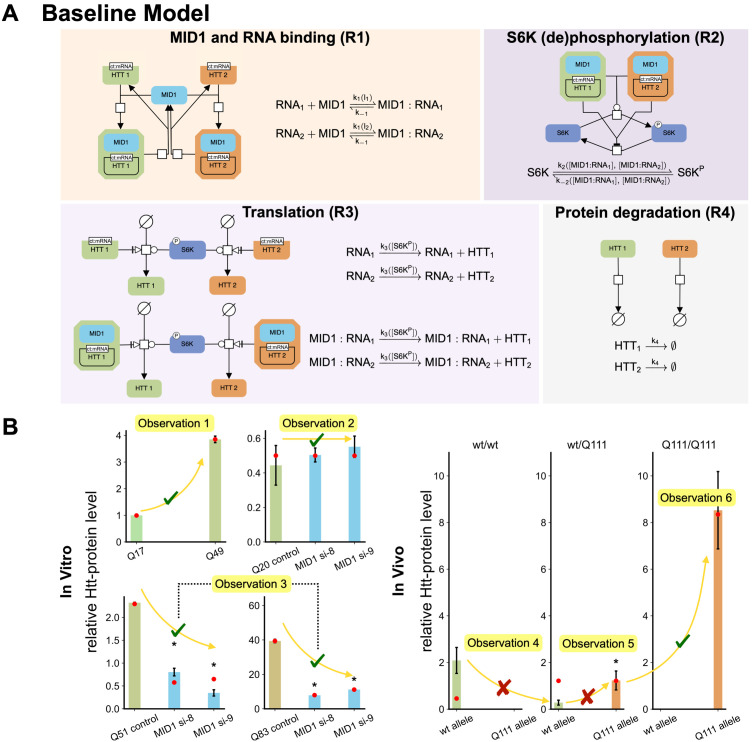
*Baseline Model*’s SBGN visualization and fitting results. **(A)** Visualization of the *Baseline Model* using the Systems Biology Graphical Notation (SBGN) language, showing the binding of MID1 and RNA has a downstream effect on S6K phosphorylation and subsequently translation. **(B)** Simulation results of the *Baseline Model* for the maximum likelihood estimate (red dot): only four out of six data observations are reproduced. The error bars represent the standard deviation of the data. An asterisk above a bar indicates a significant difference (*p* < 0.05) relative to the first bar in the subplot.

The model accounts for four classes of core reactions, which are illustrated in SBGN [29,30] (Fig 2A):

#### (R1) CAG length-dependent binding of MID1 to *Htt* RNA.

*Htt* RNA binds MID1 in a manner dependent on the CAG repeat length *q* of each allele, i.e., *q*_1_, *q*_2_. We implement this dependence upon *q* via a function *l*(*q*), which represents the length of the RNA hairpin structure to which MID1 binds. Based on *in silico* RNA secondary structure predictions [20], *l*(*q*) is approximately constant at *l*ow CAG repeat numbers and increases approximately linearly beyond a threshold repeat length (see Sec. 2. in S1 Appendix). Since MID1 is assumed to bind along this hairpin structure, a longer hairpin provides a larger binding interface and thus increases the effective association rate. We therefore model the forward binding rate by mass action kinetics with *k*_1_ proportional to *l*:


$$\begin{array}{c} {\text{RNA}}_{1}+\text{MID1}\overset{{\text{k}}_{1}(l_{1})}{\underset{{\text{k}}_{-1}}{⇌}}{\text{MID1:RNA}}_{1},\\ {\text{RNA}}_{2}+\text{MID1}\overset{{\text{k}}_{1}(l_{2})}{\underset{{\text{k}}_{-1}}{⇌}}{\text{MID1:RNA}}_{2}, \end{array}$$

where $k_{1}(l_{1})=c_{1}\cdot l_{1}(q_{1})$, and the unbinding rate $k_{-1}=c_{-1}$ is assumed to be independent of CAG repeat length. In the following text, *k* represents the reaction rate and *c* represents the reaction rate constant.

#### (R2) S6K phosphorylation modulated by MID1-bound RNA.

Binding of MID1 to *Htt* RNA promotes S6K phosphorylation and inhibits its dephosphorylation:


$$\begin{array}{c} \text{S6K}\overset{{\text{k}}_{2}}{\underset{{\text{k}}_{-2}}{⇌}}{\text{S6K}}^{\text{P}}, \end{array}$$

with the reaction rates:


$$\begin{array}{c} k_{2}(x)=c_{2}\cdot ([{MID1:RNA}_{1}]+[{MID1:RNA}_{2}]), \end{array}$$

$$\begin{array}{c} k_{-2}(x)=\frac{c_{-2}}{K_{-2}+[{MID1:RNA}_{1}]+[{MID1:RNA}_{2}]}, \end{array}$$

where *x* is the state vector and *K*_−2_ is the inhibition constant for MID1:RNA mediated suppression of S6K dephosphorylation. Since S6K is neither produced nor degraded, the total pool of S6K is conserved, i.e., $\left[S6K]+[{S6K}^{P}\right]$ remains constant. Note that the [S6K^P^] modeled here only represents the concentration level modulated by MID1. We discuss how phosphorylated S6K, modulated by MID1 or not, affects translation rate in the following reaction (R3).

#### (R3) Translation as a function of S6K^P^ abundance.

Translation occurs for both free and MID1-bound RNA and is positively regulated by phosphorylated S6K modulated by MID1:


$$\begin{array}{c} {RNA}_{1}\overset{{\text{k}}_{3}}{\to }{RNA}_{1}+{HTT}_{1},\\ {RNA}_{2}\overset{{\text{k}}_{3}}{\to }{RNA}_{2}+{HTT}_{2},\\ {MID1:RNA}_{1}\overset{{\text{k}}_{3}}{\to }{\text{MID1:RNA}}_{1}+{HTT}_{1},\\ {MID1:RNA}_{2}\overset{{\text{k}}_{3}}{\to }{\text{MID1:RNA}}_{2}+{HTT}_{2}, \end{array}$$

with the reaction rates:


$$\begin{array}{c} k_{3}(x)=c_{3}(1+\xi_{3}\cdot [{S6K}^{P}]), \end{array}$$

where *c*_3_ is the basal translation rate that includes all other sources of translational activity in the cell. For instance, *c*_3_ includes all other effects on the translation rate from phosphorylated S6K not directly modulated by MID1. We assume the effect from the S6K^P^ modulated by MID1, $c_{3}\cdot \xi_{3}\cdot [{S6K}^{P}]$, cannot be too large compared to the basal translation and constrain $\xi_{3}$ to values between 1 and 100.

#### (R4) Degradation of HTT.

The fourth reaction is first-order degradation of HTT:


$$\begin{array}{c} {\text{HTT}}_{1}\overset{{\text{k}}_{4}}{\to }\emptyset ,\;{\text{HTT}}_{2}\overset{{\text{k}}_{4}}{\to }\emptyset \end{array}$$

with the degradation rate $k_{4}=c_{4}$.

The list of reactions was translated into an ODE model. The system of ODEs governing the dynamics of the *Baseline Model* is provided in Sec. 1.1 in S1 Appendix. This model and all subsequent models admit a unique solution that remains non-negative for all *t* ≥ 0 (see Sec. 3. in S1 Appendix). The initial RNA levels are assumed to be the same and are estimated using the parameter *rna*_0_. The initial MID1 level depends on specific experiment conditions, i.e., on the presence of siRNAs (see Sec. 4. in S1 Appendix). The initial S6K level is estimated using the parameter *s*6*k*_0_. All other initial conditions are assumed to be zero.

The CAG repeat length *q* is generally known for most experimental conditions. For instance, the experimental condition Q111/Q111 means that both alleles carry 111 CAG repeats, i.e., $q_{1}=q_{2}=111$. However, for wt/Q111 and wt/wt settings, the CAG repeat length for the wt allele is not exactly known. We thus define the CAG repeat length of the wt allele in wt/Q111 and wt/wt settings to be an estimated parameter, *Q*_*wt*_ (see Sec. 5. in S1 Appendix). The reaction rate constants, e.g., *c*_1_, are also unknown parameters.

To test whether the model can explain the data (Fig 1B), the model parameters are estimated by maximum likelihood estimation (see Materials and methods for details on parameter estimation). As the data provides information about the HTT abundance in steady state, we have the observables


$$\begin{array}{c} y_{1}=s_{g}\cdot [{HTT}_{1}],\;\text{and}\;y_{2}=s_{g}\cdot [{HTT}_{2}], \end{array}$$

with western blot gel-specific scaling factors *s*_*g*_, g∈{1,2,…,5}, capturing difference in detection and normalization effects.

Parameter estimation using a state-of-the-art pipeline provides reproducible results (see Sec. 1.1 in S1 Appendix). The comparison of data and model simulations reveals that four observations are captured (Fig 2B), i.e., Observation 1, Observation 2, Observation 3, and Observation 6. However, the model fails to explain two other essential observations of the data, i.e., Observation 4 and Observation 5. These discrepancies suggest that additional molecular mechanisms—beyond the core MID1-dependent molecular interactions encoded in the *Baseline Model*—are required to fully capture the observed protein level patterns.

### Model-based analysis indicates different degradation rates for free and MID1-bound *Htt* RNA

**Fig 3:**
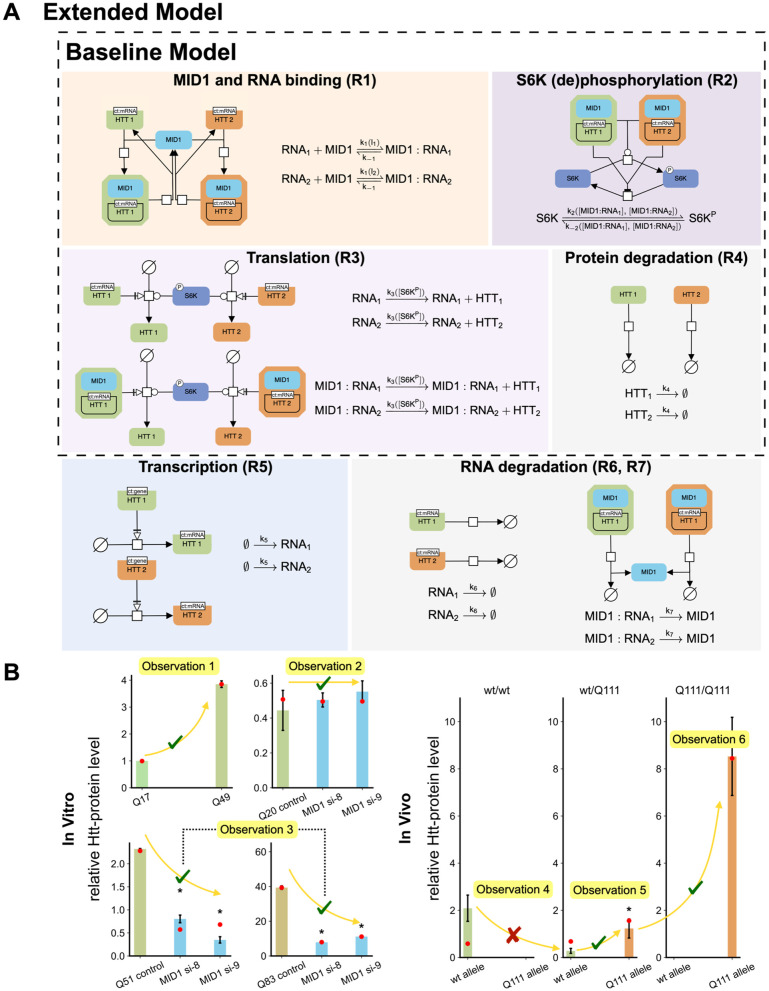
*Extended Model*’s SBGN illustration and fitting results. **(A)** *Extended Model* builds on *Baseline Model* by incorporating *Htt* RNA synthesis and degradation. **(B)** The model captures additional Observation 5 compared to the *Baseline Model*, including higher translation of the Q111 allele in heterozygous mice. The error bars represent the standard deviation of the data. An asterisk above a bar indicates a significant difference (*p* < 0.05) relative to the first bar in the subplot.

To obtain a more consistent description of the experimental data, we adjust the *Baseline Model* with additional reactions. The *Baseline Model* assumes a constant translatable RNA resource, i.e., *rna*_0_. For a dynamical system with many interacting species, this simplified assumption is, however, rarely holds. Thus, instead of assuming a fixed total *Htt* RNA level for both alleles, we now explicitly model *Htt* RNA synthesis and degradation. The *Extended Model* retains the molecular species and interactions of the *Baseline Model* but introduces three additional reactions (Fig 3A):

#### (R5) Transcription of *Htt* RNA.

New RNA for both alleles is assumed to be transcribed at a constant rate:


$$\begin{array}{c} \emptyset \overset{{\text{k}}_{5}}{\to }{RNA}_{1},\\ \emptyset \overset{{\text{k}}_{5}}{\to }{RNA}_{2}. \end{array}$$

The transcription rate for both alleles is given by $k_{5}=c_{5}$.

#### (R6) Degradation of free *Htt* RNA.

Free *Htt* RNA degrades following first-order kinetics with a constant rate:


$$\begin{array}{c} {RNA}_{1}\overset{{\text{k}}_{6}}{\to }\emptyset ,\\ {RNA}_{2}\overset{{\text{k}}_{6}}{\to }\emptyset . \end{array}$$

The degradation rate for both alleles is given by $k_{6}=c_{6}$.

#### (R7) Degradation of MID1-bound *Htt* RNA.

MID1-bound *Htt* RNA degrades following first-order kinetics, independently of free *Htt* RNA, releasing MID1:


$$\begin{array}{c} {MID1:RNA}_{1}\overset{{\text{k}}_{7}}{\to }MID1,\\ {MID1:RNA}_{2}\overset{{\text{k}}_{7}}{\to }MID1. \end{array}$$

The degradation rate for both alleles is given by $k_{7}=c_{7}$.

The initial condition, states, parameter set, and system of ODE governing the dynamics of the extended model are provided in S1 Appendix. The parameters of the model incorporating *Htt* RNA synthesis and degradation are determined using maximum likelihood estimation. The same setup as for the *Baseline Model* is applied, and the optimizer outputs indicate the optimization has converged (see Sec. 6. in S1 Appendix).

The analysis of the estimation results reveals that the *Extended Model* reproduces another important experimental observation compared to the *Baseline Model*. In particular, it correctly recapitulates Observation 5 in addition to the Observations captured by the *Baseline Model* (Fig 3B). However, the model still fails to reproduce Observation 4, where the wt allele produces a lower HTT level in wt/Q111 mice compared to in wt/wt mice.

Despite its limitations, the *Extended Model* now successfully fits Observation 5 and provides a mechanistic explanation for the higher Q111 protein level compared to the wt protein level in wt/Q111 mice. We analyze the steady state total RNA concentrations (free and MID1-bound) for both alleles and find a consistently higher total RNA level for the Q111 allele (see Sec. 7. in S1 Appendix). This asymmetry in RNA abundance, despite equal transcription rates, indicates that differences in degradation rates play a central role.

**Fig 4:**
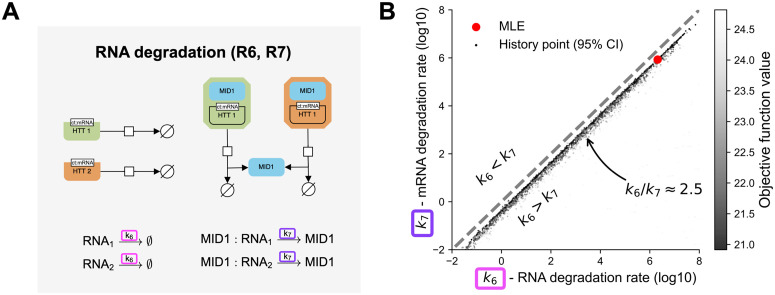
MID1 binding increases RNA stability. **(A)** Illustration of free and MID1-bound RNA degradation pathways. **(B)** Scatter plot of (*k*_6_, *k*_7_) parameter pairs (log10 scale). The maximum likelihood estimate (red) and points along the optimizer path (gray scale values according to the objective function) are shown. Only parameter vectors within the 95% confidence region, as defined via the $\chi^{2}$ threshold, are included. The median ratio across all points in the confidence region is $k_{6}/k_{7}\approx 2.5$ (linear scale).

Indeed, our analysis of the inferred parameters shows that the two degradation processes (Fig 4A) have different rate constants. The degradation rate constant of MID1-bound RNA (*c*_7_) is lower than that of free RNA (*c*_6_) (Fig 4B). Importantly, no inequality constraint is imposed on *c*_6_ and *c*_7_ in the *Extended Model*: both parameters are estimated freely over wide ranges, and the relationship *c*_6_ > *c*_7_ emerges from the data. However, the confidence intervals of *c*_6_ and *c*_7_ individually span most of the search space, indicating poor practical identifiability of the individual parameters. More broadly, the *Extended Model* exhibits wide confidence intervals for most kinetic parameters, reflecting the challenge of constraining 25 model parameters with only 48 measurements at steady state representing 14 distinct experimental conditions (see S1 Table and Sec. 8. in S1 Appendix). Despite this, the two-dimensional confidence region reveals that the ratio of *c*_6_ and *c*_7_ is consistently estimated at $c_{6}/c_{7}\approx 2.5$: free RNA degrades approximately 2.5 times faster than MID1-bound RNA. Thus, while the individual rate constants are not identifiable, the key conclusion that MID1 binding reduces the degradation rate is robust across the entire confidence region. This finding implies that MID1 binding not only enhances translation efficiency via S6K phosphorylation but also stabilizes *Htt* RNA by protecting it from degradation.

### Experimental data validates MID1 mediated protection of *Htt* RNA against degradation

**Fig 5:**
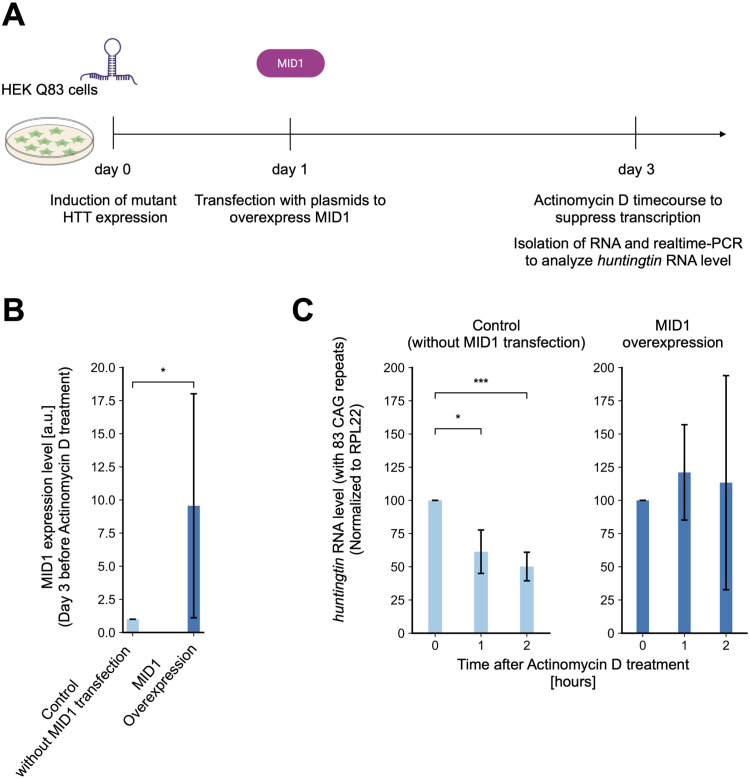
Experimental data verifies the model prediction that MID1 increases RNA stability and thereby the abundance of *Htt* RNA relative to wt. **(A)** Experimental setup. **(B)** MID1 level comparison under control (without MID1 transfection) and MID1 overexpression conditions on day 3 (*n* = 6 biological replicates per condition). **(C)** Relative *Htt* RNA (with 83 CAG repeats) level within 2 hours under control (without MID1 transfection) and MID1 overexpression conditions (*n* = 6 biological replicates per condition). The error bars represent the standard deviation of the data. Asterisks indicate significant differences relative to the 0h time point within each condition (Bonferroni-corrected paired comparisons; * represents *p* < 0.05, ^***^ represent *p* < 0.001). Between-condition differences were assessed using Mann-Whitney U tests (1h: *p* = 0.008; 2h: *p* = 0.041).

To validate the model-based prediction of an altered degradation rate, we conduct a new experiment and assess how *Htt* RNA degrades under two conditions: a control condition (without MID1 transfection) and a MID1 overexpression condition (Fig 5A). After the establishment of the cell culture, including the induction of a *Htt* allele with 83 CAG repeats (Q83) in the cell and transfection with plasmids coding for MID1, cells are treated with Actinomycin D to inhibit transcription. Subsequently, the relative RNA levels are monitored using qPCR.

The assessment of the MID1 levels three days after plasmid transfection confirms a substantial increase compared to the control condition (Fig 5B). The difference in MID1 levels results in different *Htt* RNA decay dynamics (Fig 5C). Under the control condition (without MID1 transfection), the *Htt* RNA level is reduced by 50% after two hours, indicating a half-life time of approximately 2 hours. Under the MID1 overexpression condition, the *Htt* RNA levels are stable over the same time interval, implying a substantially longer half-life. The difference between conditions is significant at both times (Mann-Whitney U test, *p* = 0.008 at 1 h, *p* = 0.041 at 2 h). Individual replicate values are provided in S2 Table.

The experimental data provides a validation of our model-based predictions, illustrating the significant functional role of MID1 in regulating RNA stability.

### Clustering and local signaling explain observed HTT levels

The validation experiments show that the *Extended Model* can provide valuable, testable hypotheses. Yet, the model does not recapitulate Observation 4. As the underlying cause of this limitation is unclear, we employ model-based hypothesis testing.

Since the increased stability of MID1-bound RNA has been experimentally confirmed, we incorporate this finding as a constraint (*k*_7_ ≤ *k*_6_) that enforces it in all subsequent models as a starting point for the model-based hypothesis testing. To account for the constraint, we reformulate the degradation reaction as follows:

#### (R6_R_) Degradation of free *Htt* RNA.

Free *Htt* RNA degrades at a constant rate:


$$\begin{array}{c} {RNA}_{1}\overset{{\text{k}}_{6,\text{R}}}{\to }\emptyset ,\\ {RNA}_{2}\overset{{\text{k}}_{6,\text{R}}}{\to }\emptyset . \end{array}$$

The degradation rate for both alleles is given by $k_{6,R}=\xi_{6}k_{7}$, where $1\leq \xi_{6}$. The parameter $\xi_{6}$ encodes the stability difference between free and MID1-bound RNA.

In the following part, we explore three models developed upon the *Extended Model*, each introducing an additional mechanism to explain Observation 4 (the wt allele produces lower HTT levels in wt/Q111 mice than in wt/wt mice), which neither the *Baseline Model* nor the *Extended Model* could reproduce.

In the wt/Q111 setting, the mutant allele binds MID1 more strongly than the wt allele, driving stronger S6K^P^ activity. If translation responds linearly to S6K^P^, this difference in activity would not be sufficient to suppress wt allele translation below its level in the wt/wt setting. However, if translation depends nonlinearly on S6K^P^, for instance, through a threshold-like response, then the redistribution of S6K^P^ activity toward the mutant allele could markedly reduce wt translation efficiency. Such a nonlinearity might arise as translation is a multi-step process and might depend nonlinearly on the abundance of S6K^P^. We therefore assess a Hill-type formulation, where translation efficiency increases slowly at low S6K^P^ concentrations but rises sharply beyond a certain threshold. The *Extended Model with Nonlinear Translation* retains the molecular species and interactions from the *Extended Model* but utilizes a Hill-type reaction rate for reaction (R3):

#### (R3_H_) Translation as a function of S6K^P^ abundance.

Translation occurs for both free and MID1-bound RNA and is positively regulated by phosphorylated S6K:


$$\begin{array}{c} {RNA}_{1}\overset{{\text{k}}_{3,\text{H}}}{\to }{RNA}_{1}+{HTT}_{1},\\ {RNA}_{2}\overset{{\text{k}}_{3,\text{H}}}{\to }{RNA}_{2}+{HTT}_{2},\\ {MID1:RNA}_{1}\overset{{\text{k}}_{3,\text{H}}}{\to }{\text{MID1:RNA}}_{1}+{HTT}_{1},\\ {MID1:RNA}_{2}\overset{{\text{k}}_{3,\text{H}}}{\to }{\text{MID1:RNA}}_{2}+{HTT}_{2}, \end{array}$$

with the reaction rates:


$$\begin{array}{c} k_{3,H}(x)=c_{3}\left(1+\xi_{3}\cdot \frac{[{{S6K}^{P}]}^{n_{3}}}{K_{3}^{n_{3}}+[{{S6K}^{P}]}^{n_{3}}}\right). \end{array}$$

A second possibility we explore is a *l*-dependent clustering mechanism of MID1-bound RNA. If MID1-bound RNA is spatially segregated into local clusters, the stronger MID1 binding of the mutant allele would concentrate S6K^P^ activity locally, depleting the global pool available to the wt allele and thereby suppressing its translation. Such spatial segregation is consistent with evidence that cytoplasmic RNA is often non-uniformly distributed, becoming enriched in discrete sites such as membraneless condensates [31], TIS granules [32], or translation factories [33], where local translation rates could differ markedly from those in the surrounding cytosol. A spatial enrichment can arise through a variety of mechanisms, including physical clustering of RNA (often associated with RNA granules) [31,34], targeted localization to translation-permissive compartments [32], or co-translational recruitment of related RNA through nascent chain interactions [35,36].

**Fig 6:**
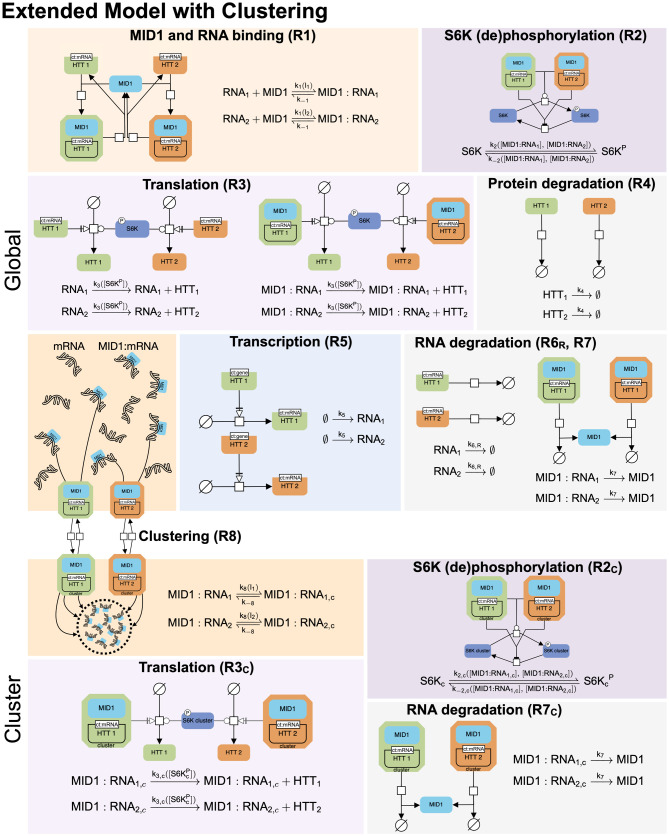
SBGN illustration of the *Extended Model with Clustering.* MID1:RNA complexes form a local cluster that selectively modulates nearby S6K phosphorylation and translation activity.

To capture this possibility, we model “clusters” in a generalized sense as any localized subpopulation of MID1-bound RNA, regardless of the precise mechanism underlying their enrichment, whose translation can be selectively modulated by locally elevated S6K^P^ activity. Such compartmentalized regulation enables precise, context-dependent protein synthesis and may account for the nonlinear dynamics and allele-specific effects observed in the experimental data. The *Extended Model with Clustering* incorporates spatial structure implicitly through localized translation regulation. It assumes that MID1-bound *Htt* RNA can aggregate into spatially restricted molecular clusters, where they exert local influence on S6K phosphorylation inside the cluster. This localized S6K activation selectively enhances translation of clustered RNA without affecting global translation rates. Outside the cluster, translation is assumed to proceed as in the *Extended Model*. The *Extended Model with Clustering* introduces several new reactions (Fig 6), and expands the state vector to include the cluster-localized species $\left[{MID1:RNA}_{1,c}\right]$, $\left[{MID1:RNA}_{2,c}\right]$, $\left[{S6K}_{c}\right]$, and $\left[S6K_{c}^{P}\right]$. The additional and modified reactions are:

#### (R8) Cluster association and dissociation.

MID1-bound RNA can reversibly join clusters:


$$\begin{array}{c} {MID1:RNA}_{1}\overset{{\text{k}}_{8}({\text{l}}_{1})}{\underset{{\text{k}}_{-8}}{⇌}}{MID1:RNA}_{1,c},\\ {MID1:RNA}_{2}\overset{{\text{k}}_{8}({\text{l}}_{2})}{\underset{{\text{k}}_{-8}}{⇌}}{MID1:RNA}_{2,c}. \end{array}$$

We consider a CAG repeat length dependent association rate for cluster entry, i.e., $k_{8}(l)=c_{8}\cdot l$, and a constant dissociation rate $k_{-8}=c_{-8}$. In Sec. 9.1 in S1 Appendix, we show that models lacking CAG repeat length dependence (i.e., $k_{8}(l)\equiv c_{8}$) fail to recapitulate the observations, suggesting that *l* dependence is a possible mechanism consistent with all observations.

#### (R2_C_) Local S6K phosphorylation.

Within the cluster, S6K is phosphorylated in proportion to the amount of MID1-bound RNA present:


$$\begin{array}{c} {\text{S6K}}_{c}\overset{{\text{k}}_{2,\text{C}}}{\underset{{\text{k}}_{-2,\text{C}}}{⇌}}{\text{S6K}}_{c}^{\text{P}}. \end{array}$$

The rates follow the same functional form as outside the cluster,


$$\begin{array}{c} k_{2,C}(x)=c_{2}\cdot ([{MID1:RNA}_{1,c}]+[{MID1:RNA}_{2,c}]), \end{array}$$

$$\begin{array}{c} k_{-2,C}(x)=\frac{c_{-2}}{K_{-2}+[{MID1:RNA}_{1,c}]+[{MID1:RNA}_{2,c}]}, \end{array}$$

but are not identical: the S6K phosphorylation rate outside the cluster, which remains the same as in the *Baseline Model* and the *Extended Model*, depends on the MID1-bound RNA outside the cluster, whereas the cluster rate depends on the cluster-localized MID1-bound RNA.

#### (R3_C_) Translation inside clusters as a function of S6K^P^ abundance.

MID1:RNA complexes in the clusters are translated in an S6K^P^-dependent manner,


$$\begin{array}{c} {MID1:RNA}_{1,c}\overset{{\text{k}}_{3,\text{C}}}{\to }{MID1:RNA}_{1,c}+{HTT}_{1},\\ {MID1:RNA}_{2,c}\overset{{\text{k}}_{3,\text{C}}}{\to }{MID1:RNA}_{2,c}+{HTT}_{2}. \end{array}$$

with rate:


$$\begin{array}{c} k_{3,C}(x)=c_{3}(1+\xi_{3}\cdot [S6K_{c}^{P}]). \end{array}$$

The translation outside the cluster remains the same as in *Baseline Model* and in the *Extended Model*.

#### (R7_C_) Degradation of the MID1:RNA in the cluster.

Both global and cluster-associated MID1:RNA species degrade at the same MID1-dependent rate $k_{7}=c_{7}$, distinct from the degradation of unbound RNA.

As the two mechanisms, nonlinear translation and clustering, are not exclusive, we also consider the *Extended Model with Clustering and Nonlinear Translation*. The model is based on *Extended Model with Clustering*, with the translation outside the clusters changed to (R3_H_) and the translation inside is defined as the following:

#### (R3_C, H_) Translation inside clusters as a function of S6K^P^ abundance.

MID1:RNA complexes in the clusters are translated in an S6K^P^-dependent manner,


$$\begin{array}{c} {MID1:RNA}_{1,c}\overset{{\text{k}}_{3,\text{C},\text{H}}}{\to }{MID1:RNA}_{1,c}+{HTT}_{1},\\ {MID1:RNA}_{2,c}\overset{{\text{k}}_{3,\text{C},\text{H}}}{\to }{MID1:RNA}_{2,c}+{HTT}_{2}. \end{array}$$

with rate:


$$\begin{array}{c} k_{3,C,H}(x)=c_{3}\left(1+\xi_{3}\cdot \frac{[{{S6K}^{P}]}^{n_{3}}}{K_{3}^{n_{3}}+[{{S6K}^{P}]}^{n_{3}}}\right). \end{array}$$

The ODE systems governing the dynamics of all three models are provided in Sec. 1. in S1 Appendix. All models respect allele symmetry: swapping the labeling of allele 1 and allele 2 does not change the model output.

**Fig 7:**
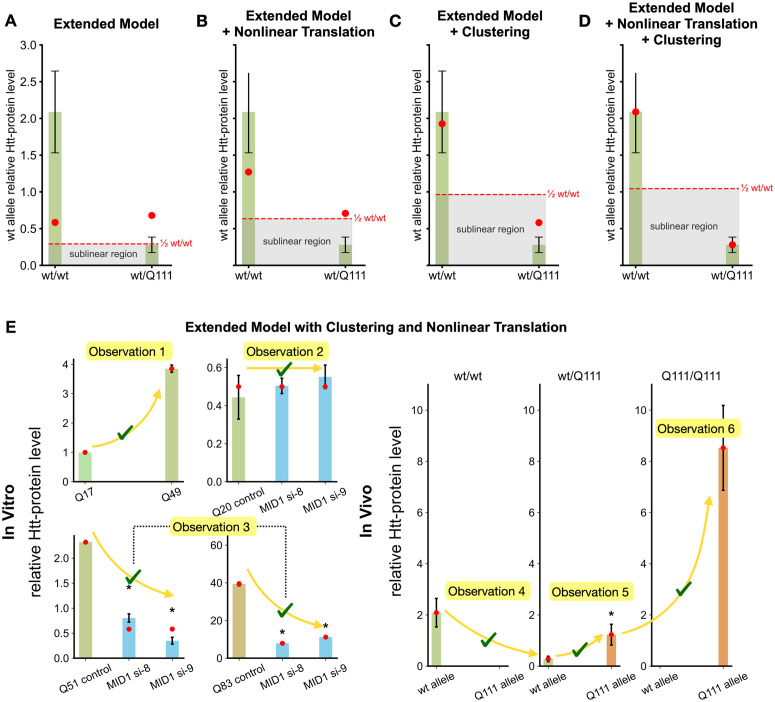
Comparison of model predictions for wild-type allele–derived HTT levels in *wt/wt* and *wt/Q111* settings and fitting results of the *Extended Model with Clustering and Nonlinear Translation.* The HTT levels of the wt allele are indicated for (A) the *Extended Model*, (B) the *Extended Model with Nonlinear Translation*, (C) the *Extended Model with Clustering*, (D) the *Extended Model with Clustering and Nonlinear Translation*. Compared to *Extended Model*, the other three models all improve the fitting regarding the difference in wt allele HTT level between in wt/wt mice and wt/Q111 mice, but only the models with clustering reproduce the sublinear suppression. The red dashed line represents half of the wt allele HTT level of the simulation in the wt/wt setting, and the gray area represents the sublinear HTT level region that is below the half. **(E)** The *Extended Model with Clustering and Nonlinear Translation* successfully reproduces all observations. The error bars represent the standard deviation of the data. An asterisk above a bar indicates a significant difference (*p* < 0.05) relative to the first bar in the subplot.

The analysis of the estimation results reveals that the three new models with additional mechanisms progressively deepen the suppression of the wt HTT level in the wt/Q111 setting (Fig 7B-7D) compared to the *Extended Model* (Fig 7A). Such suppression is achieved when the wt allele HTT level in the wt/Q111 setting is less than half of the level in the wt/wt setting (the region below the half line is thus called the sublinear region). The threshold is at half of the HTT level in wt/wt because wt/Q111 has one instead of two wt alleles. The *Extended Model* is unable to reproduce such suppression, and in fact, the wt allele in the wt/Q111 setting has a higher HTT level. In the model with only nonlinear translation, the simulated wt HTT level in the wt/Q111 setting is still a bit higher than in the wt/wt setting and lies slightly above the half line, outside of the sublinear region. The model with clustering alone produces a downward shift, but combining both non-linearity and clustering yields the strongest suppression effect among the three models, leading the wt HTT level well inside the sublinear region.

Looking at full fitting results of all three models, only the models with clustering can capture all observations (Fig 7E), while the model with only nonlinear translation does not fully reproduce Observation 4 (see Sec. 9. in S1 Appendix). Although *Extended Model with Clustering* also captures all observations, only the simulation results of *Extended Model with Clustering and Nonlinear Translation* are within the standard deviation of the data (Fig 7E).

To quantitatively compare the performance of all models we consider in this work, we calculate the Negative Log-likelihood (NLLH), the Akaike Information Criterion (AIC), its small-sample corrected version (AICc), and the Bayesian Information Criterion (BIC) for each model (Table 1). The *Extended Model with Clustering and Nonlinear Translation* achieves the best fit, as indicated by the lowest NLLH. However, this improved fit requires more free parameters compared to the *Extended Model with Clustering*, which leads to higher complexity penalties and thus worse AIC values. Because our dataset is small (|𝒟|=48), these penalties are further amplified in AICc and BIC, resulting in the *Baseline Model* scoring best under these criteria.

**Table 1:** Overview of the performance of different model topologies. NLLH, AIC, AICc and BIC values are reported – with lower values indicating better performance – as well as the number of model parameters ($n_{\theta }$), all of which are estimated via maximum likelihood estimation. Furthermore, it is indicated with a checkmark (✓) if the model reproduces the observations outlined in the text.

Metric / Observation	Baseline Model	Extended Model	Extended Model + NT	Extended Model + Clustering	Extended Model + Clustering + NT
**NLLH**	22.45	20.92	18.23	15.04	**14.02**
**AIC**	90.90	91.83	90.47	**86.08**	88.04
**AICc**	**136.90**	150.92	166.07	171.55	197.45
**BIC**	**133.94**	138.61	140.99	138.47	144.18
$n_{\theta }$	23	25	27	28	30
Observation 1	✓	✓	✓	✓	✓
Observation 2	✓	✓	✓	✓	✓
Observation 3	✓	✓	✓	✓	✓
Observation 4				✓	✓
Observation 5		✓	✓	✓	✓
Observation 6	✓	✓	✓	✓	✓

### Simulation study demonstrates how nonlinear translation and clustering help capture all experimentally reported observations

As demonstrated in the last section, the *Extended Model with Clustering and Nonlinear Translation* can reproduce all observations, and its simulation results are within the standard deviation of the data. Specifically, the model can reproduce Observation 4 (the wt allele produces lower HTT levels in wt/Q111 compared to wt/wt mice) and Observation 6 (Q111/Q111 mice show a supralinear increase in HTT levels from the Q111 allele).

**Fig 8:**
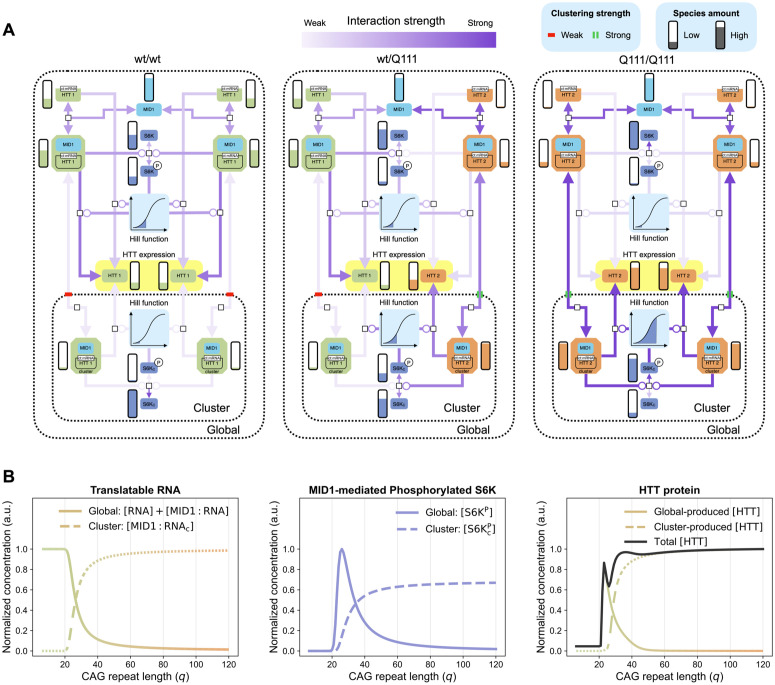
*Extended Model with Clustering and Nonlinear Translation*: simulation study under the three *in vivo* experimental conditions. **(A)** Comparison of steady state amounts of model species (bars) and interaction strengths in the cluster compartment (purple-gradient arrows) across wt/wt, wt/Q111, and Q111/Q111 settings. CAG repeat length–dependent clustering strength is shown for wild-type (red bar) and mutant (green pass) alleles. Amounts and interactions are from steady state simulations at the maximum likelihood estimate of the model; the Hill function of R3_C,H_ is shown at the cluster center. **(B)** Steady state simulations for $q_{1}=q_{2}\in [6,120]$: left, translatable RNA in global and cluster pools; middle, phosphorylated S6K in both pools; right, HTT levels in each pool and in total. The color gradient represents the CAG repeat length in *Htt* RNA and the Q length in HTT. Curves in each subplot are normalized by the highest value of any quantity in the respective subplot for visual clarity.

To understand how the model captures the allele-specific HTT level patterns, we examine how CAG repeat length shapes the steady state of the model species across the three *in vivo* settings (Fig 8A). As CAG repeat length increases from wt/wt to Q111/Q111, stronger MID1 binding shifts more RNA into MID1-bound and subsequently clustered states. This redistribution has two opposing effects on S6K^P^: the global pool decreases, as more MID1:RNA is sequestered into clusters and unavailable to drive global S6K phosphorylation, while the cluster pool increases due to locally elevated MID1:RNA concentrations.

These opposing effects, combined with the nonlinear Hill-type translation response, explain both key observations. For Observation 4, in the wt/Q111 setting, the wt allele resides predominantly in the global environment, while the mutant allele moves into the cluster, causing a reduction of S6K^P^. The Hill-type response amplifies this effect into a reduction in wt translation, reproducing the sublinear wt HTT level. For Observation 6, in the Q111/Q111 setting, both alleles are strongly clustered, and the locally elevated $\left[S6K_{c}^{P}\right]$ drives a sharp increase in cluster translation via the Hill function. The combined loss of global translation and gain of cluster translation produces the observed supralinear increase in Q111 HTT levels. Thus, CAG repeat length-dependent clustering coupled to a nonlinear translation response converts differences in MID1 binding into the allele-specific HTT level patterns observed across the three experimental settings.

To go beyond comparing only the limited number of experimental settings, we systematically explored how different CAG repeat lengths influence the steady state abundance of model species in the *Extended Model with Clustering and Nonlinear Translation*. Specifically, we conduct a simulation study with CAG repeat length between 6 and 120, i.e., $q_{1}=q_{2}$ and $q_{1},q_{2}\in [6,120]$. These simulations reveal that the model predicts that the patterns observed across the three *in vivo* settings (Fig 8A) are part of a continuous transition, in which translation shifts from being dominated by the global pool at low *q* to being dominated by the cluster pool at high *q* (Fig 8B, right). Such a shift is caused by two components, translatable RNA and MID1-mediated phosphorylated S6K. Specifically, as CAG repeat length increases, we observe a decrease of global translatable resources and an increase of cluster translatable resources as the resources move into the cluster (Fig 8B, left). Interestingly, [S6K^P^] does not follow a monotone decrease and has a sharp increase just after *q* = 20 CAG repeats (Fig 8B, middle). The sharp increase is due to the shape of the *l* function (see Sec. 10. in S1 Appendix). Together, those two components drive the HTT curve, global-produced or cluster-produced.

## Discussion

The interaction between RNA and RBP plays a pivotal role in post-transcriptional gene regulation, governing processes such as RNA stability, localization, and translation. Disruptions to these interactions have been increasingly recognized as pathogenic mechanisms, with HD being an example [37]. Motivated by experimental data showing a CAG repeat length dependent increase in protein level, we construct a sequence of ODE models to bridge the molecular biochemistry of MID1 binding and S6K phosphorylation with the emergent allele-specific HTT level patterns observed *in vitro* and *in vivo*. The progression—from a minimal *Baseline Model*, through an explicit RNA turnover model (*Extended Model*), to its extensions incorporating nonlinear translation and clustering—allows us to pinpoint which mechanistic ingredients are required to reproduce the full experimental phenotype.

The *Baseline Model*, which encodes only the core known reactions, fails to account for more nuanced observations such as allele-specific HTT level asymmetries. The *Extended Model* extended the *Baseline Model* by adding RNA synthesis and decay. While still incomplete, the model suggests that differences in *Htt* RNA stability, with and without MID1 binding, could explain the preferential translation of the Q111 allele. Indeed, prior work has shown that MID1, besides stimulating translation, may affect RNA stability [38]. This prediction is experimentally confirmed in our work: elevated MID1 levels substantially increase the half-life of mutant *Htt* RNA compared to wt. This agreement between model and experiment establishes the *Extended Model* as not only explanatory but also predictive, offering mechanistic insights with direct biological validation.

The prolonged availability of *Htt* RNA due to increased stability through MID1 binding can substantially impact protein synthesis dynamics, potentially exacerbating the accumulation of mutant HTT, a hallmark of HD pathology. Thus, our findings not only reveal a previously unrecognized aspect of RNA regulation by MID1 but also offer valuable insights into the underlying molecular mechanisms contributing to disease progression. Identifying and characterizing this novel MID1-dependent stabilization pathway represents an important step toward developing targeted therapeutic interventions aimed at modulating mutant HTT level through MID1 interaction.

Neither the *Baseline Model* nor the *Extended Model* could explain the suppressed wt HTT level in wt/Q111 (Observation 4), indicating that additional mechanisms may be at play. Firstly, we treat translation downstream of S6K^P^ as cooperative (Hill-type) in the *Extended Model with Nonlinear Translation*. This modification improves the fit to Observation 4 and suggests that S6K^P^ may drive cooperative, threshold-like translational control.

Another extension replaces the well-mixed assumption with a spatially structured description: MID1-bound RNAs can form a local cluster, where S6K phosphorylation and translation are regulated by the local concentrations of MID1:RNA and S6K. This *Extended Model with Clustering* reflects accumulating evidence that many cytoplasmic RNAs reside in sub-cytoplasmic microdomains (e.g., TIS granules/rough endoplasmic reticulum) where translation differs from the surrounding cytosol [32].

The concept of a localized cluster with RBP has been studied as a mechanism for protein synthesis [33]. Our study extends this concept specifically to MID1-mediated clustering of *Htt* RNA. Indeed, MID1 has a known ability to anchor RNA to the microtubule cytoskeleton, facilitating such spatial organization [24,38]. Furthermore, as MID1 preferentially binds to RNA with expanded CAG repeats, it is plausible that such clustering is CAG repeat length dependent. This dependence has been shown in other work [39]. These findings suggest that subcellular localization and compartmentalized signaling may affect translation efficiency in the context of CAG repeat expansion. As such, disrupting RNA-protein clustering may present a promising therapeutic avenue for modulating allele-specific translation in HD and related disorders.

While the model prediction of CAG repeat length dependent MID1-bound RNA clustering is plausible, direct experimental validation would be required to distinguish clustering from alternative mechanisms such as local S6K activation at individual MID1:RNA complexes. Single-molecule RNA FISH [40] could reveal whether mutant *Htt* RNAs co-localize in discrete cytoplasmic foci in a CAG repeat length-dependent manner, and SunTag-based translation imaging [41] could additionally determine whether translation events are spatially clustered or dispersed. Ideally, the quantitative dependence on CAG-repeat number and MID1 levels should be assessed to recapitulate differences between patients. Such studies would offer critical insights into the spatial architecture of translation regulation in HD.

Finally, to compare the performance of all models, we compute information criteria such as AIC, AICc, and BIC. The *Baseline Model* achieves the best AICc and BIC scores, as these criteria penalize the additional parameters of the more complex models. However, the *Baseline Model* does not reproduce all observations. The *Extended Model with Clustering* and the *Extended Model with Clustering and Nonlinear Translation* achieve substantially lower NLLHs and are the only models that reproduce all observations, though at the cost of increased model complexity. These models therefore represent plausible mechanistic frameworks that can account for all observations, though the limited dataset does not allow us to draw definitive conclusions.

In addition to nonlinear translation and clustering, another non-exclusive driver of the more-than-doubling of HTT level in Q111/Q111 could be somatic CAG-repeat expansion. In HD, CAG repeat numbers can increase over time within individual neurons, especially in the striatum, creating mosaic populations with effective repeat lengths well above 111 [42]. Although not explicitly modeled here, for steady state data, this mechanism can be incorporated by estimating a higher-than-111 CAG repeat length.

Although the *Extended Model with Clustering and Nonlinear Translation* captures the key experimental trends, it remains a simplification. We currently represent a single effective cluster, whereas cells may contain multiple, compositionally distinct clusters that could shape translation in parallel. Likewise, we assume one MID1 per RNA, even though expanded *Htt* RNA could recruit multiple MID1 molecules, potentially amplifying clustering and introducing additional cooperativity not captured here. Moreover, some model assumptions lack direct experimental validation. For instance, several reaction rates, such as free RNA degradation, are assumed to be independent of CAG repeat length, as we are not aware of experimental evidence for such dependencies. Future experiments could clarify whether such dependencies exist and guide a more specific model formulation.

Beyond these structural assumptions, inference is further limited by data availability: while the dataset [20] comprises 48 measurements, these correspond to only 14 distinct experimental conditions. Of the 23–30 estimated parameters, 10 are noise and scaling parameters, leaving 13–20 kinetic parameters to be constrained by the 14 experimental conditions. The 5 noise parameters are directly informed by the replicates within each condition. Furthermore, the wt CAG length in the wt/wt and wt/Q111 cohorts was not reported, requiring inference of *Q*_*wt*_ and adding uncertainty to parameter estimates. Indeed, profile likelihood analysis (see Sec. 8. in S1 Appendix) confirms that the majority of kinetic parameters are non-identifiable across all models. This limited identifiability cautions against favoring one mechanism over another, and we therefore consider all three *Extended Model* with extra components as possible explanations. Future studies with richer time- and dose-response data, explicit reporting of wt repeat lengths, and model extensions (e.g., multiple clusters or multivalent MID1 binding) are needed to improve identifiability and test robustness.

Our integrative modeling and experimental study uncovers a novel mechanistic role for MID1 in promoting both the stability and localized translation of mutant *Htt* RNA through spatial clustering. These findings not only provide insight into how RBP regulates pathogenic RNA behavior in HD but also highlight the critical role of subcellular localization in shaping translation outcomes. By challenging the traditional assumption of a spatially uniform intracellular environment, our work opens new directions for investigating compartmentalized post-transcriptional regulation and lays a foundation for future therapeutic strategies targeting RNA-protein clustering dynamics in neurodegenerative diseases.

## Materials and methods

### Experimental data

We consider previously published experimental data (Fig 2B) [20]. A description of the experimental protocols for this published data is in the original publications [20].

For the new validation experiment we conducted in this study, HEK293 cells stably expressing huntingtin exon1 with 83 CAG repeats in a Tet-off system [43] were seeded in poly-L-lysine coated 12 well plates at a density of 2.5 × 10^5^ cells per well. Washing off doxycycline induced the expression of huntingtin exon1. Cells were then cultured for 24 hours and transfected with a plasmid (pCMVTag2A-MID1 [24]) to overexpress MID1. After 48 hours of incubation, the actinomycin D time course was started. For this purpose, 1 μ l actinomycin D (5 mg/ml; Merck) was added per 1 ml medium, and the cells were harvested after 0 h, 1 h, or 2 h. Cells were washed with PBS and harvested using a cell scraper, followed by total RNA isolation using the Monarch Total RNA Miniprep Kit (NEB). cDNA was synthesized using the TaqMan reverse transcription reagents kit (Applied Biosystems), and real-time PCR was carried out using the qPCRBIO SyGreen Mix (PCRBiosystems). The sequences of primers are specified in Table 2.

**Table 2:** Primer sequences used in this study.

Primer	Sequence
RPL22-for	TGACATCCGAGGTGCCTTTC
RPL22-rev	GTTAGCAACTACGCGCAACC
mutHTTexon1-Flag-for	CGCGGCCCCGAATT
mutHTTexon1-Flag-rev	TCTTTGTAGTCCATGGTGGTTCA
MID1-for	TTGGAATGGTCCATGAATTAAGG
MID1-rev	CAAACTAGAACCAATGCCAGAGTT

### Mathematical formulation

We model the interaction among all the molecules using systems of ODEs. These ODE models are of the form:


$$\begin{array}{c} \frac{dx}{dt}=f(x(t,\theta ,u),\theta ,u),\;x(0)=x_{0}(\theta ,u) \end{array}$$

with state vector $x(t)\in {\mathbb{R}}^{n_{x}}$ at time *t*, parameter vector $\theta \in {\mathbb{R}}^{n_{\theta }}$, and input vec*t*or $u\in {\mathbb{R}}^{n_{u}}$ that encodes the experimental conditions. The vector field $f:{\mathbb{R}}^{n_{x}}\times {\mathbb{R}}^{n_{\theta }}\times {\mathbb{R}}^{n_{u}}\to {\mathbb{R}}^{n_{x}}$ encodes the dynamics of the process and is constructed to be Lipschitz continuous. The function $x_{0}:{\mathbb{R}}^{n_{\theta }}\times {\mathbb{R}}^{n_{u}}\to {\mathbb{R}}^{n_{x}}$ defines the initial condition. By *n*_*x*_, $n_{\theta }$, and *n*_*u*_ we denote the numbers of state variables, unknown mechanistic parameters, and known input variables, respectively.

Model observables are the measurable model quantities. They are defined as functions of the state vector *x* and the parameter vector θ


$$\begin{array}{c} y=h(x(t,\theta ,u),\theta ), \end{array}$$

with $h:{\mathbb{R}}^{n_{x}}\times {\mathbb{R}}^{n_{\theta }}\to {\mathbb{R}}^{n_{y}}$. For notational simplicity, we assume that there is only one measurable quantity, i.e., $n_{y}=1$. When the measurements are relative, meaning that they provide data only in a relative form rather than as absolute quantities, it is necessary to introduce scaling factors that scale the observables to the data. For an assumed additive and normally distributed measurement noise, i.e., $\epsilon_{i,j,k}\sim 𝒩(0,\sigma_{i,k}^{2})$, the measurement-to-observable relationship is given by:


$$\begin{array}{c} y_{i,j,k}^{m}=s_{i,k}\cdot h(x(t_{k},\theta ,u_{i}),\theta )+\epsilon_{i,j,k}, \end{array}$$

with experimental conditions of each experiment $i\in \{1,2,\ldots ,n_{e}\}$, repetition index $j\in \{1\ldots n_{i}\}$, time point $k\in \{1,2,\ldots ,n_{t}\}$, and noise standard deviation $\sigma_{i,k}$. By *n*_*e*_, *n*_*i*_, and *n*_*t*_ we denote the number of experiments, the number of data replicates and the number of time points. A dataset is defined as the set of all measurements,


$$\begin{array}{c} 𝒟={\left\{{\left\{{\left\{(t_{k},y_{i,j,k}^{m})\right\}}_{i=1}^{n_{e}}\right\}}_{j=1}^{n_{i}}\right\}}_{k=1}^{n_{t}}. \end{array}$$

Unknown model parameters (θ,s,σ) are inferred from the measured data using maximum likelihood estimation. The likelihood function of observing the experimental data 𝒟 given parameters θ, *s*, and σ is given by


$$\begin{array}{c} {ℒ}_{𝒟}(\theta ,s,\sigma )=p(𝒟|\theta ,s,\sigma )=\prod_{i=1}^{n_{e}}\prod_{j=1}^{n_{i}}\prod_{k=1}^{n_{t}}p(y_{i,j,k}^{m}|s_{i,k}\cdot h(x(t_{k},\theta ,u_{i}),\theta ),\sigma_{i,k}), \end{array}$$

in which the likelihood can be separated into a product of conditional probabilities of individual measurements because of the assumed pairwise independence of all measurements. For a Gaussian noise model, the conditional probability of observing an individual measurement $y_{i,j,k}^{m}$ given the observable value of the model $s_{i,k}\cdot h(x(t_{k},\theta ,u_{i}),\theta )$ and the noise level $\sigma_{i,k}$ is given by


$$\begin{array}{c} p(y_{i,j,k}^{m}|s_{i,k}\cdot h(x(t_{k},\theta ,u_{i}),\theta ),\sigma_{i,k})=\frac{1}{\sqrt{2\pi \sigma_{i,k}^{2}}}\exp\left(-\frac{1}{2}\frac{{\left(y_{i,j,k}^{m}-s_{i,k}\cdot h(x(t_{k},\theta ,u_{i})\right)}^{2}}{\sigma_{i,k}^{2}}\right). \end{array}$$

Instead of directly maximizing ${ℒ}_{𝒟}$, it is equivalent and numerically often preferable to minimize the negative log-likelihood $J=-\log{ℒ}_{𝒟}$. Assuming Gaussian noise, this objective function is given by


$$\begin{array}{cc} J(\theta ,s,\sigma ) & =-\sum_{i=1}^{n_{e}}\sum_{j=1}^{n_{i}}\sum_{k=1}^{n_{t}}\log p(y_{i,j,k}^{m}|y(t_{k},\theta ,u_{i}),\sigma_{i,k})\\ & =\sum_{i=1}^{n_{e}}\sum_{j=1}^{n_{i}}\sum_{k=1}^{n_{t}}\frac{1}{2}\left(\log\left(2\pi \sigma_{i,k}^{2}\right)+\frac{{\left(y_{i,j,k}^{m}-s_{i,k}\cdot h(x(t_{k},\theta ,u_{i})\right)}^{2}}{\sigma_{i,k}^{2}}\right). \end{array}$$

For better convergence and efficiency properties [44–46], the unknown model parameters are estimated hierarchically: scaling *s* and noise parameters σ of the models are estimated in the inner optimization problem, and all other model parameters θ are estimated in the outer optimization problem. The hierarchical optimization problem is given by


$$\begin{array}{cc} \hat{\theta }=\underset{\theta }{\text{arg}\min}\; & J(\theta ,\hat{s},\hat{\sigma }),\text{ subject to }\theta_{lb}\leq \theta \leq \theta_{ub}\\ \text{where } & \left\{ \begin{array}{c} \left\{\hat{s},\hat{\sigma }\}=\underset{s,\sigma }{\text{arg}\min}\;J(\theta ,s,\sigma \right)\\ \text{subject to }\;\sigma_{lb}\leq \sigma \leq \sigma_{ub}\\ \;\;\;\;\;\;s_{lb}\leq s\leq s_{ub}, \end{array}\right. \end{array}$$

in which the inner optimization problem can be solved analytically [46].

The selection of the bounds for the unknown model parameters (θ,s,σ) is outlined in Sec. 11. in S1 Appendix.

### Steady state computation

A steady state of an ODE model $\dot{x}=f(x,\theta ,u)$ is defined as a state vector x^*^ satisfying


$$\begin{array}{c} f(x^{*},\theta ,u)=0, \end{array}$$

i.e., all time derivatives vanish. Numerically, steady states can be obtained in different ways. One common approach is to directly solve the nonlinear system f(x,θ,u)=0 using a Newton-type root-finding method, which requires evaluating and factorizing the Jacobian ∂f/∂x. Alternatively, the system can be integrated forward in time until the norm of f(x,θ,u) falls below a predefined tolerance, indicating proximity to a steady state. In practice, simulation tools, including the one used in this study, alternate between these steady state calculation approaches for increased robustness [47]. In all cases, suitable convergence criteria (e.g., relative and absolute tolerances) must be specified to determine when the system is sufficiently close to steady state.

### Model selection criteria

We employ the Akaike Information Criterion (AIC) [48], its small-sample corrected version (AICc) [49], and the Bayesian Information Criterion (BIC) [50] as model selection metrics to assess the trade-off between goodness-of-fit and model complexity. These criteria are defined as:


$$\begin{array}{c} AIC\;=\;2n_{\theta }-2\ln(ℒ(\hat{\theta },\hat{s},\hat{\sigma })), \end{array}$$

$$\begin{array}{c} \;=\;2n_{\theta }+2J(\hat{\theta },\hat{s},\hat{\sigma }), \end{array}$$

$$\begin{array}{c} AICc\;=\;AIC+\frac{2n_{\theta }^{2}+2n_{\theta }}{|D|-n_{\theta }-1}, \end{array}$$

$$\begin{array}{c} BIC\;=n_{\theta }\ln(|D|)-2\ln(ℒ(\hat{\theta },\hat{s},\hat{\sigma })), \end{array}$$

$$\begin{array}{c} \;=n_{\theta }\ln(|D|)+2J(\hat{\theta },\hat{s},\hat{\sigma }), \end{array}$$

where $ℒ(\hat{\theta },\hat{s},\hat{\sigma })$ is the maximum likelihood value, and |𝒟| is the number of data points.

### Uncertainty quantification

Here, we define what it means for a parameter vector to lie in a confidence region of a certain significance. This is done using the likelihood-ratio test, in which the corresponding test statistic is defined as


$$\begin{array}{c} \Lambda (\theta )=-2\log\left(\frac{{ℒ}_{𝒟}(\theta )}{{ℒ}_{𝒟}(\hat{\theta })}\right)=2(J(\theta )-J(\hat{\theta })). \end{array}$$ (1)

In the asymptotic case of a large number of data points, the test statistic converges to a chi-square distribution $\chi_{\text{df}}^{2}$ with $\text{df}=n_{\theta }$ degrees of freedom (see [51] for more details). Then the confidence region of significance α is defined as


$$\begin{array}{c} {\text{CR}}^{\alpha }=\{\theta |\Lambda (\theta )\leq \Delta_{\alpha }\}, \end{array}$$ (2)

in which $\Delta_{\alpha }$ is the α-th percentile of the $\chi_{n_{\theta }}^{2}$ distribution. The one-dimensional projection of a confidence region for a specific parameter $\theta_{m}$ of the parameter vector θ is defined as the Confidence Interval (CI) of that parameter:


$$\begin{array}{c} {\text{CI}}_{\theta_{m}}^{\alpha }=\{\theta_{m}|\Lambda (\theta )\leq \Delta_{\alpha }^{1}\}, \end{array}$$ (3)

in which $\Delta_{\alpha }^{1}$ is the α-th percentile of the $\chi_{1}^{2}$ distribution with one degree of freedom. Prediction uncertainty quantification can then be carried out by propagating parameter uncertainty using the CR to model predictions.

### Implementation

We use PEtab [52] to define the parameter estimation problems and SBML [53] for standardized representations of the mathematical models. We create the SBML models using Tellurium’s Antimony [54]. Parameter optimization and uncertainty analysis are implemented using pyPESTO 0.5.6 [55], wherein we use AMICI 0.33.0 [47] for simulation and steady state calculation, and Fides 0.7.8 [56] for optimization. Each model is estimated using gradient-based multi-start local optimization with 50000 randomly sampled starting points. As is commonly done, most parameters are estimated on the logarithmic scale to uniformly explore parameter orders of magnitude [57].

## Supporting information

S1 Appendix Supplementary sections, figures, and tables. Contains detailed model description, optimization setup, and complementary results.
(PDF)

S1 Table Parameter estimates and confidence intervals. Maximum likelihood estimates and 95% profile likelihood confidence interval bounds for all parameters across all models.
(XLSX)

S2 Table Experimental replicate values for Fig 5. Individual replicate measurements for MID1 expression levels (Fig 5B) and *Htt* RNA levels under control and MID1 overexpression conditions (Fig 5C).
(XLSX)
