# Supplementary material for: Data-driven model reveals increased stability of CAG-expanded huntingtin RNA due to MID1 binding
Source: PLoS Comput Biol. 2026 Jun 2;22(6):e1014342. doi: 10.1371/journal.pcbi.1014342 (PMC13252851; doi:10.1371/journal.pcbi.1014342)
Supplement: S1 Appendix — Contains detailed model description, optimization setup, and complementary results. (PDF) [file pcbi.1014342.s001.pdf]

Supplementary information - Data-driven model reveals increased stability of CAG-expanded *huntingtin* RNA due to MID1 binding

Yuhong Liu [1,2], Annika Reisbitzer [3], Domagoj Dorešić [1,2], Jan Hasenauer [1,2*], Sybille Krauß [3*], and Tatjana Tchumatchenko [4*]

[1] Life and Medical Sciences (LIMES) Institute, University of Bonn, Bonn, Germany
[2] Bonn Center for Mathematical Life Sciences, University of Bonn, Bonn, Germany
[3] Institute of Biology, University of Siegen, Siegen, Germany
[4] Institute of Experimental Epileptology and Cognition Research, University of Bonn Medical Center, Bonn, Germany
[*] These authors contributed equally

Contents

1 ODE systems and initial conditions **3**
1.1 Baseline Model 3
1.2 Extended Model 5
1.3 Extended Model with Nonlinear Translation Rate 7
1.4 Extended Model with Clustering (l-dependent) 8
1.5 Extended Model with Clustering and Nonlinear Translation (l-dependent) . . 11
1.6 Extended Model with Clustering (non-l-dependent) 11
1.7 Extended Model with Clustering and Nonlinear Translation Rate (non-l-dependent) 11

2 Activation function, l, as a function of CAG repeat length q **12**

3 Existence, uniqueness, and non-negative invariance of ODE solutions proof 12

4 Experimental Condition 14

5 Optimized Q_{wt} distribution 15

6 Convergence of optimization 17

7 Steady-state RNA and MID1:RNA level comparison between wt and Q111 alleles 18

8 Parameter identifiability analysis 19

9 Comparison of fitting results of all models 31

9.1 l dependence is necessary for clustering rate 33

10 Effect of the Hill function on translation dynamics across CAG repeat lengths 34

11 Complete Parameter Table 37

1 ODE systems and initial conditions

Here we present in detail the dynamical system, the initial condition, and the parameter vector of all models.

1.1 Baseline Model

The *Baseline Model* considers the four core reactions (R1-R4) mentioned in the main manuscript, which involve the following state vector:

$$x = \begin{pmatrix} [\mathrm{RNA}_1] \\ [\mathrm{RNA}_2] \\ [\mathrm{MID1}] \\ [\mathrm{MID1} : \mathrm{RNA}_1] \\ [\mathrm{MID1} : \mathrm{RNA}_2] \\ [\mathrm{S6K}] \\ [\mathrm{S6K}^{\mathrm{P}}] \\ [\mathrm{HTT}_1] \\ [\mathrm{HTT}_2] \end{pmatrix}.$$

We assume the initial condition, i.e., x_0, is as follows:

$$x_0 = \begin{pmatrix} [\mathrm{RNA}_1]_0 \\ [\mathrm{RNA}_2]_0 \\ [\mathrm{MID1}]_0 \\ [\mathrm{MID1} : \mathrm{RNA}_1]_0 \\ [\mathrm{MID1} : \mathrm{RNA}_2]_0 \\ [\mathrm{S6K}]_0 \\ [\mathrm{S6K}^{\mathrm{P}}]_0 \\ [\mathrm{HTT}_1]_0 \\ [\mathrm{HTT}_2]_0 \end{pmatrix} = \begin{pmatrix} rna_0 \cdot allele_1 \\ rna_0 \cdot allele_2 \\ \exp(si8_{\mathrm{effect}} \cdot si8_{\mathrm{flag}} + si9_{\mathrm{effect}} \cdot si9_{\mathrm{flag}}) \\ 0 \\ 0 \\ s6k_0 \\ 0 \\ 0 \\ 0 \end{pmatrix},$$

Here, rna_0, $s6k_0$, $si8_{\mathrm{effect}}$ and $si9_{\mathrm{effect}}$ are parameters to be estimated, while $allele_1$, $allele_2$, $si8_{\mathrm{flag}}$ and $si9_{\mathrm{flag}}$ are part of the experimental condition (see Sec. 4). In the *Baseline Model*, we assume the initial RNA level is a constant, contrary to the *Extended Model* (see Sec. 1.2). The binary values of $allele_1$ and $allele_2$ indicate whether the experiment is performed *in vitro* (e.g., $allele_1 = 1$ and $allele_2 = 0$) or *in vivo* ($allele_1 = allele_2 = 1$). In some experiments, MID1 is knocked down to assess its effect on the HTT level using small interfering RNA (siRNA) techniques-specifically with si8 or si9. The flags $si8_{\mathrm{flag}}$ and $si9_{\mathrm{flag}}$ indicate when a specific type of knockdown is applied, and the corresponding effects are captured by the

parameters $si8_{\text{effect}}$ and $si9_{\text{effect}}$. Because the intracellular concentration of S6K is unknown, the initial condition $s6k_0$ is also treated as an estimated parameter.

Given the reactions (R1-R4), we can write down the ODE for each species according to the reactions in which it participates. Combining these equations yields the complete ODE system, $\forall t \in [0, \infty)$

$$
\begin{aligned}
\frac{d[\mathrm{RNA_1}]}{dt} &= -k_1(l_1)[\mathrm{RNA_1}][\mathrm{MID1}] \\
&\quad + k_{-1}[\mathrm{MID1:RNA_1}], \\
\frac{d[\mathrm{RNA_2}]}{dt} &= -k_1(l_2)[\mathrm{RNA_2}][\mathrm{MID1}] \\
&\quad + k_{-1}[\mathrm{MID1:RNA_2}], \\
\frac{d[\mathrm{MID1}]}{dt} &= -k_1(l_1)[\mathrm{RNA_1}][\mathrm{MID1}] - k_1(l_2)[\mathrm{RNA_2}][\mathrm{MID1}] \\
&\quad + k_{-1}[\mathrm{MID1:RNA_1}] + k_{-1}[\mathrm{MID1:RNA_2}] \\
\frac{d[\mathrm{MID1:RNA_1}]}{dt} &= k_1(l_1)[\mathrm{RNA_1}][\mathrm{MID1}] \\
&\quad - k_{-1}[\mathrm{MID1:RNA_1}], \\
\frac{d[\mathrm{MID1:RNA_2}]}{dt} &= k_1(l_2)[\mathrm{RNA_2}][\mathrm{MID1}] \\
&\quad - k_{-1}[\mathrm{MID1:RNA_2}], \\
\frac{d[\mathrm{S6K}]}{dt} &= k_2([\mathrm{MID1:RNA_1}],[\mathrm{MID1:RNA_2}])[\mathrm{S6K}] \\
&\quad - k_{-2}([\mathrm{MID1:RNA_1}],[\mathrm{MID1:RNA_2}])[\mathrm{S6K^P}], \\
\frac{d[\mathrm{S6K^P}]}{dt} &= -k_2([\mathrm{MID1:RNA_1}],[\mathrm{MID1:RNA_2}])[\mathrm{S6K}] \\
&\quad + k_{-2}([\mathrm{MID1:RNA_1}],[\mathrm{MID1:RNA_2}])[\mathrm{S6K^P}], \\
\frac{d[\mathrm{HTT_1}]}{dt} &= k_3([\mathrm{S6K^P}])[\mathrm{RNA_1}] + k_3([\mathrm{S6K^P}])[\mathrm{MID1:RNA_1}] \\
&\quad - k_4[\mathrm{HTT_1}], \\
\frac{d[\mathrm{HTT_2}]}{dt} &= k_3([\mathrm{S6K^P}])[\mathrm{RNA_2}] + k_3([\mathrm{S6K^P}])[\mathrm{MID1:RNA_2}] \\
&\quad - k_4[\mathrm{HTT_2}],
\end{aligned}
$$

where,

$$\begin{aligned}
k_1(l_\zeta) &= c_1 \cdot l_\zeta(q_\zeta), \zeta \in \{1,2\}, \\
k_{-1} &= c_{-1}, \\
k_2([\mathrm{MID1}:\mathrm{RNA}_1],[\mathrm{MID1}:\mathrm{RNA}_2]) &= c_2 \cdot ([\mathrm{MID1}:\mathrm{RNA}_1]+[\mathrm{MID1}:\mathrm{RNA}_2]), \\
k_{-2}([\mathrm{MID1}:\mathrm{RNA}_1],[\mathrm{MID1}:\mathrm{RNA}_2]) &= \frac{c_{-2}}{K_{-2}+[\mathrm{MID1}:\mathrm{RNA}_1]+[\mathrm{MID1}:\mathrm{RNA}_2]}, \\
k_3([\mathrm{S6K^P}]) &= c_3 \cdot (1+\xi_3 \cdot [\mathrm{S6K^P}]), \\
k_4 &= c_4.
\end{aligned}$$

Based on the above ODE system, we point out that the system is symmetric with respect to RNA_1 and RNA_2, meaning switching the two variables would not change the model dynamics.

We combine all parameters used in this work in one table (see Sec. 11), and the parameters that are part of the *Baseline Model* can be written in the vector form:

$$\begin{aligned}
\theta = (&c_1, c_{-1}, c_2, c_{-2}, K_{-2}, c_3, \xi_3, c_4, Q_{\mathrm{wt}}, rna_0, s6k_0, \\
&si8_{\mathrm{effect}}, si9_{\mathrm{effect}}, s_1, s_2, s_3, s_4, s_5, \sigma_1, \sigma_2, \sigma_3, \sigma_4, \sigma_5)^\top.
\end{aligned}$$

The scaling parameters $(s_1, \ldots, s_5)$ are introduced in the main text and discussed in more detail in Sec. 11. The details on noise parameters $(\sigma_1, \ldots, \sigma_5)$ are included in Sec. 11.

1.2 Extended Model

Since the additional three reactions (R5-R7) do not add additional species, the state vector of *Extended Model* stays the same as in the *Baseline Model*. However, the initial condition x_0 is now changed to:

$$x_0 = \begin{pmatrix} [\mathrm{RNA}_1]_0 \\ [\mathrm{RNA}_2]_0 \\ [\mathrm{MID1}]_0 \\ [\mathrm{MID1}:\mathrm{RNA}_1]_0 \\ [\mathrm{MID1}:\mathrm{RNA}_2]_0 \\ [\mathrm{S6K}]_0 \\ [\mathrm{S6K^P}]_0 \\ [\mathrm{HTT}_1]_0 \\ [\mathrm{HTT}_2]_0 \end{pmatrix} = \begin{pmatrix} 0 \\ 0 \\ \exp(si8_{\mathrm{effect}} \cdot si8_{\mathrm{flag}} + si9_{\mathrm{effect}} \cdot si9_{\mathrm{flag}}) \\ 0 \\ 0 \\ s6k_0 \\ 0 \\ 0 \\ 0 \end{pmatrix}.$$

In the *Baseline Model*, we assume the same RNA initial conditions, $[\mathrm{RNA}_\zeta]_0 = s6k_0$, across all experimental conditions. Because there is no RNA degradation reaction and $[\mathrm{MID1}:\mathrm{RNA}_\zeta]_0 = 0$, the $[\mathrm{RNA}_\zeta]+[\mathrm{MID1}:\mathrm{RNA}_\zeta]$ should be conserved to rna_0 under all experimental conditions

and the four reactions. In the *Extended Model*, such conservation does not exist any more because two reactions, RNA production and RNA degradation, are added. For simplicity, we assume the initial condition of RNA is 0.

Given the reactions (R1-R7), the complete ODE system is as follows, $\forall t \in [0, \infty)$:

$$
\begin{aligned}
\frac{d[\mathrm{RNA}_1]}{dt} &= -k_1(l_1)[\mathrm{RNA}_1][\mathrm{MID1}] \\
&\quad + k_{-1}[\mathrm{MID1} : \mathrm{RNA}_1] \\
&\quad + k_5 \cdot allele_1 - k_6[\mathrm{RNA}_1], \\
\frac{d[\mathrm{RNA}_2]}{dt} &= -k_1(l_2)[\mathrm{RNA}_2][\mathrm{MID1}] \\
&\quad + k_{-1}[\mathrm{MID1} : \mathrm{RNA}_2] \\
&\quad + k_5 \cdot allele_2 - k_6[\mathrm{RNA}_2], \\
\frac{d[\mathrm{MID1}]}{dt} &= -k_1(l_1)[\mathrm{RNA}_1][\mathrm{MID1}] - k_1(l_2)[\mathrm{RNA}_2][\mathrm{MID1}] \\
&\quad + k_{-1}[\mathrm{MID1} : \mathrm{RNA}_1] + k_{-1}[\mathrm{MID1} : \mathrm{RNA}_2] \\
&\quad + k_7([\mathrm{MID1} : \mathrm{RNA}_1] + [\mathrm{MID1} : \mathrm{RNA}_2]), \\
\frac{d[\mathrm{MID1} : \mathrm{RNA}_1]}{dt} &= k_1(l_1)[\mathrm{RNA}_1][\mathrm{MID1}] \\
&\quad - k_{-1}[\mathrm{MID1} : \mathrm{RNA}_1] \\
&\quad - k_7[\mathrm{MID1} : \mathrm{RNA}_1], \\
\frac{d[\mathrm{MID1} : \mathrm{RNA}_2]}{dt} &= k_1(l_2)[\mathrm{RNA}_2][\mathrm{MID1}] \\
&\quad - k_{-1}[\mathrm{MID1} : \mathrm{RNA}_2] \\
&\quad - k_7[\mathrm{MID1} : \mathrm{RNA}_2], \\
\frac{d[\mathrm{S6K}]}{dt} &= k_2([\mathrm{MID1} : \mathrm{RNA}_1], [\mathrm{MID1} : \mathrm{RNA}_2])[\mathrm{S6K}] \\
&\quad - k_{-2}([\mathrm{MID1} : \mathrm{RNA}_1], [\mathrm{MID1} : \mathrm{RNA}_2])[\mathrm{S6K}^{\mathrm{P}}], \\
\frac{d[\mathrm{S6K}^{\mathrm{P}}]}{dt} &= -k_2([\mathrm{MID1} : \mathrm{RNA}_1], [\mathrm{MID1} : \mathrm{RNA}_2])[\mathrm{S6K}] \\
&\quad + k_{-2}([\mathrm{MID1} : \mathrm{RNA}_1], [\mathrm{MID1} : \mathrm{RNA}_2])[\mathrm{S6K}^{\mathrm{P}}], \\
\frac{d[\mathrm{HTT}_1]}{dt} &= k_3([\mathrm{S6K}^{\mathrm{P}}])[\mathrm{RNA}_1] + k_3([\mathrm{S6K}^{\mathrm{P}}])[\mathrm{MID1} : \mathrm{RNA}_1] \\
&\quad - k_4[\mathrm{HTT}_1], \\
\frac{d[\mathrm{HTT}_2]}{dt} &= k_3([\mathrm{S6K}^{\mathrm{P}}])[\mathrm{RNA}_2] + k_3([\mathrm{S6K}^{\mathrm{P}}])[\mathrm{MID1} : \mathrm{RNA}_2] \\
&\quad - k_4[\mathrm{HTT}_2],
\end{aligned}
$$

where,

$$\begin{aligned}
k_1(l_\zeta) &= c_1 \cdot l_\zeta, \zeta \in \{1,2\},\\
k_{-1} &= c_{-1},\\
k_2([\text{MID1}:\text{RNA}_1],[\text{MID1}:\text{RNA}_2]) &= c_2 \cdot ([\text{MID1}:\text{RNA}_1]+[\text{MID1}:\text{RNA}_2]),\\
k_{-2}([\text{MID1}:\text{RNA}_1],[\text{MID1}:\text{RNA}_2]) &= \frac{c_{-2}}{K_{-2}+[\text{MID1}:\text{RNA}_1]+[\text{MID1}:\text{RNA}_2]},\\
k_3([\text{S6K}^\text{P}]) &= c_3 \cdot (1+\xi_3 \cdot [\text{S6K}^\text{P}]),\\
k_4 &= c_4,\\
k_5 &= c_5 \cdot allele_\zeta,\\
k_6 &= c_6,\\
k_7 &= c_7.
\end{aligned}$$

Notice that the experimental condition indicating *in vitro* or *in vivo* experiment, $allele_\zeta$, is now part of the transcription rate, $k_5 \cdot allele_\zeta$. The control with $allele_\zeta$ on whether one allele produces RNA or not would also subsequently control whether other reactions of that allele happen or not.

Because of the extra three reactions, three parameters are added and rna_0 is removed. The new parameter vector is:

$$\begin{aligned}
\theta = (&c_1, c_{-1}, c_2, c_{-2}, K_{-2}, c_3, \xi_3, c_4, c_5, c_6, c_7, Q_\text{wt}, s6k_0,\\
&si8_\text{effect}, si9_\text{effect}, s_1, s_2, s_3, s_4, s_5, \sigma_1, \sigma_2, \sigma_3, \sigma_4, \sigma_5)^\top,
\end{aligned}$$

1.3 Extended Model with Nonlinear Translation Rate

The state vector, initial condition, and ODE systems of the *Extended Model with Nonlinear Translation* are the same as in the *Extended Model*, with only k_3 changed to $k_{3,H}$ (R3_H):

$$k_{3,H}([\text{S6K}^\text{P}]) = c_3 \left(1 + \xi_3 \cdot \frac{[\text{S6K}^\text{P}]^{\text{n}_3}}{K_3^{n_3} + [\text{S6K}^\text{P}]^{\text{n}_3}}\right),$$

and k_6 changed to $k_{6,R} = \xi_6 \cdot k_7$, where $1 \le \xi_6$ (R6_R). Due to the change in these two functions, the parameter vector is updated to include n_3, K_3, ξ_6:

$$\begin{aligned}
\theta = (&c_1, c_{-1}, c_2, c_{-2}, K_{-2}, c_3, \xi_3, n_3, K_3, c_4, c_5, c_6, c_7, \xi_6, Q_\text{wt}, s6k_0, si8_\text{effect},\\
&si9_\text{effect}, s_1, s_2, s_3, s_4, s_5, \sigma_1, \sigma_2, \sigma_3, \sigma_4, \sigma_5)^\top,
\end{aligned}$$

1.4 Extended Model with Clustering (l-dependent)

The *Extended Model with Clustering* (l-dependent) shares the same reactions (R1-R7) with the *Extended Model*, with one reaction modified ($R6_R$) and four new reactions (R8, $R2_C$, $R3_C$, $R7_C$) inside the cluster. The additional reactions introduce four additional states inside the cluster. The state vector is as follows:

$$x = \begin{pmatrix} [\mathrm{RNA}_1] \\ [\mathrm{RNA}_2] \\ [\mathrm{MID1}] \\ [\mathrm{MID1} : \mathrm{RNA}_1] \\ [\mathrm{MID1} : \mathrm{RNA}_2] \\ [\mathrm{S6K}] \\ [\mathrm{S6K}^{\mathrm{P}}] \\ [\mathrm{MID1} : \mathrm{RNA}_{1,\mathrm{c}}] \\ [\mathrm{MID1} : \mathrm{RNA}_{2,\mathrm{c}}] \\ [\mathrm{S6K}_{\mathrm{c}}] \\ [\mathrm{S6K}_{\mathrm{c}}^{\mathrm{P}}] \\ [\mathrm{HTT}_1] \\ [\mathrm{HTT}_2] \end{pmatrix},$$

with initial condition x_0 being:

$$x_0 = \begin{pmatrix} [\mathrm{RNA}_1]_0 \\ [\mathrm{RNA}_2]_0 \\ [\mathrm{MID1}]_0 \\ [\mathrm{MID1} : \mathrm{RNA}_1]_0 \\ [\mathrm{MID1} : \mathrm{RNA}_2]_0 \\ [\mathrm{S6K}]_0 \\ [\mathrm{S6K}^{\mathrm{P}}]_0 \\ [\mathrm{MID1} : \mathrm{RNA}_{1,\mathrm{c}}]_0 \\ [\mathrm{MID1} : \mathrm{RNA}_{2,\mathrm{c}}]_0 \\ [\mathrm{S6K}_{\mathrm{c}}]_0 \\ [\mathrm{S6K}_{\mathrm{c}}^{\mathrm{P}}]_0 \\ [\mathrm{HTT}_1]_0 \\ [\mathrm{HTT}_2]_0 \end{pmatrix} = \begin{pmatrix} 0 \\ 0 \\ \exp(si8_{\mathrm{effect}} \cdot si8_{\mathrm{flag}} + si9_{\mathrm{effect}} \cdot si9_{\mathrm{flag}}) \\ 0 \\ 0 \\ s6k_0 \\ 0 \\ 0 \\ 0 \\ s6k_0 \cdot \xi_{[\mathrm{S6K_c}]_0} \\ 0 \\ 0 \\ 0 \end{pmatrix}.$$

Notice the extra parameter $\xi_{[\mathrm{S6K_c}]_0}$. Because the cluster forms as more MID1:RNA is being produced, the concentration of S6K inside and outside the cluster can be dynamic. However, as we only have steady-state measurements, we assume the conserved pool of S6K and $\mathrm{S6K_c}$ are constant. We define the constant ratio between $[\mathrm{S6K_c}]_0$ and $[\mathrm{S6K}]_0$ as $\xi_{[\mathrm{S6K_c}]_0}$.

Given all the reactions, the complete ODE system is as follows, $\forall t \in [0, \infty)$:

$$
\begin{aligned}
\frac{d[\mathrm{RNA_1}]}{dt} &= -k_1(l_1)[\mathrm{RNA_1}][\mathrm{MID1}] \\
&\quad + k_{-1}[\mathrm{MID1:RNA_1}] \\
&\quad + k_5 - k_{6,R}[\mathrm{RNA_1}], \\
\frac{d[\mathrm{RNA_2}]}{dt} &= -k_1(l_2)[\mathrm{RNA_2}][\mathrm{MID1}] \\
&\quad + k_{-1}[\mathrm{MID1:RNA_2}] \\
&\quad + k_5 - k_{6,R}[\mathrm{RNA_2}], \\
\frac{d[\mathrm{MID1}]}{dt} &= -k_1(l_1)[\mathrm{RNA_1}][\mathrm{MID1}] - k_1(l_2)[\mathrm{RNA_2}][\mathrm{MID1}] \\
&\quad + k_{-1}[\mathrm{MID1:RNA_1}] + k_{-1}[\mathrm{MID1:RNA_2}] \\
&\quad + k_7([\mathrm{MID1:RNA_1}] + [\mathrm{MID1:RNA_2}]) \\
&\quad + k_7([\mathrm{MID1:RNA_{1,c}}] + [\mathrm{MID1:RNA_{2,c}}]), \\
\frac{d[\mathrm{MID1:RNA_1}]}{dt} &= k_1(l_1)[\mathrm{RNA_1}][\mathrm{MID1}] \\
&\quad - k_{-1}[\mathrm{MID1:RNA_1}] \\
&\quad - k_7[\mathrm{MID1:RNA_1}] \\
&\quad - k_8(l_1)[\mathrm{MID1:RNA_1}] + k_{-8}[\mathrm{MID1:RNA_{1,c}}], \\
\frac{d[\mathrm{MID1:RNA_2}]}{dt} &= k_1(l_2)[\mathrm{RNA_2}][\mathrm{MID1}] \\
&\quad - k_{-1}[\mathrm{MID1:RNA_2}] \\
&\quad - k_7[\mathrm{MID1:RNA_2}] \\
&\quad - k_8(l_2)[\mathrm{MID1:RNA_2}] + k_{-8}[\mathrm{MID1:RNA_{2,c}}], \\
\frac{d[\mathrm{S6K}]}{dt} &= k_2([\mathrm{MID1:RNA_1}], [\mathrm{MID1:RNA_2}])[\mathrm{S6K}] \\
&\quad - k_{-2}([\mathrm{MID1:RNA_1}], [\mathrm{MID1:RNA_2}])[\mathrm{S6K^P}], \\
\frac{d[\mathrm{S6K^P}]}{dt} &= -k_2([\mathrm{MID1:RNA_1}], [\mathrm{MID1:RNA_2}])[\mathrm{S6K}] \\
&\quad + k_{-2}([\mathrm{MID1:RNA_1}], [\mathrm{MID1:RNA_2}])[\mathrm{S6K^P}], \\
\frac{d[\mathrm{MID1:RNA_{1,c}}]}{dt} &= k_8(l_1)[\mathrm{MID1:RNA_1}] - k_{-8}[\mathrm{MID1:RNA_{1,c}}] - k_7[\mathrm{MID1:RNA_{1,c}}], \\
\frac{d[\mathrm{MID1:RNA_{2,c}}]}{dt} &= k_8(l_2)[\mathrm{MID1:RNA_2}] - k_{-8}[\mathrm{MID1:RNA_{2,c}}] - k_7[\mathrm{MID1:RNA_{2,c}}], \\
\frac{d[\mathrm{S6K_c}]}{dt} &= k_{2,C}([\mathrm{MID1:RNA_{1,c}}], [\mathrm{MID1:RNA_{2,c}}])[\mathrm{S6K_c}] \\
&\quad - k_{-2,C}([\mathrm{MID1:RNA_{1,c}}], [\mathrm{MID1:RNA_{2,c}}])[\mathrm{S6K_c^P}],
\end{aligned}
$$

$$
\begin{aligned}
\frac{d[\mathrm{S6K_c^P}]}{dt} &= -k_{2,C}([\mathrm{MID1:RNA_{1,c}}],[\mathrm{MID1:RNA_{2,c}}])[\mathrm{S6K_c}] \\
&+ k_{-2,C}([\mathrm{MID1:RNA_{1,c}}],[\mathrm{MID1:RNA_{2,c}}])[\mathrm{S6K_c^P}], \\
\frac{d[\mathrm{HTT_1}]}{dt} &= k_3([\mathrm{S6K^P}])[\mathrm{RNA_1}] + k_3([\mathrm{S6K^P}])[\mathrm{MID1:RNA_1}] \\
&+ k_{3,C}([\mathrm{S6K_c^P}])[\mathrm{MID1:RNA_{1,c}}] \\
&- k_4[\mathrm{HTT_1}], \\
\frac{d[\mathrm{HTT_2}]}{dt} &= k_3([\mathrm{S6K^P}])[\mathrm{RNA_2}] + k_3([\mathrm{S6K^P}])[\mathrm{MID1:RNA_2}] \\
&+ k_{3,C}([\mathrm{S6K_c^P}])[\mathrm{MID1:RNA_{2,c}}] \\
&- k_4[\mathrm{HTT_2}],
\end{aligned}
$$

where,

$$
\begin{aligned}
k_1(l_\zeta) &= c_1 \cdot l_\zeta, \zeta \in \{1,2\}, \\
k_{-1} &= c_{-1}, \\
k_2([\mathrm{MID1:RNA_1}],[\mathrm{MID1:RNA_2}]) &= c_2 \cdot ([\mathrm{MID1:RNA_1}] + [\mathrm{MID1:RNA_2}]), \\
k_{-2}([\mathrm{MID1:RNA_1}],[\mathrm{MID1:RNA_2}]) &= \frac{c_{-2}}{K_{-2} + [\mathrm{MID1:RNA_1}] + [\mathrm{MID1:RNA_2}]}, \\
k_{2,C}([\mathrm{MID1:RNA}_{1,c}],[\mathrm{MID1:RNA}_{2,c}]) &= c_2 \cdot ([\mathrm{MID1:RNA}_{1,c}] + [\mathrm{MID1:RNA}_{2,c}]), \\
k_{-2,C}([\mathrm{MID1:RNA}_{1,c}],[\mathrm{MID1:RNA}_{2,c}]) &= \frac{c_{-2}}{K_{-2} + [\mathrm{MID1:RNA}_{1,c}] + [\mathrm{MID1:RNA}_{2,c}]}, \\
k_3([\mathrm{S6K^P}]) &= c_3 \cdot (1 + \xi_3 \cdot [\mathrm{S6K^P}]), \\
k_{3,C}([\mathrm{S6K_c^P}]) &= c_3 \cdot (1 + \xi_3 \cdot [\mathrm{S6K_c^P}]), \\
k_4 &= c_4, \\
k_5 &= c_5 \cdot allele_\zeta, \\
k_{6,R} &= \xi_6 \cdot c_7, \\
k_7 &= c_7, \\
k_8((l_\zeta) &= c_8 \cdot l_\zeta, \\
k_{-8} &= c_{-8}.
\end{aligned}
$$

Notice that k_2 and $k_{2,C}$ (same for k_{-2} and $k_{-2,C}$ or k_3 and $k_{3,C}$) share the same parameters, but the inputs of the two functions are different.

Due to the extra reactions, the parameter vector is now as follows:

$$
\begin{aligned}
\theta = (&c_1, c_{-1}, c_2, c_{-2}, K_{-2}, c_3, \xi_3, c_4, c_5, c_6, c_7, \xi_6, c_8, c_{-8}, Q_{\mathrm{wt}}, s6k_0, \\
&\xi_{[\mathrm{S6K_c}]_0}, si8_{\mathrm{effect}}, si9_{\mathrm{effect}}, s_1, s_2, s_3, s_4, s_5, \sigma_1, \sigma_2, \sigma_3, \sigma_4, \sigma_5)^\top.
\end{aligned}
$$

1.5 Extended Model with Clustering and Nonlinear Translation (l-dependent)

The *Extended Model with Clustering and Nonlinear Translation* (l-dependent) shares the same state vector, initial condition, and ODE system of the *Extended Model with Clustering (l-dependent)*, with only the k_3 changed to $k_{3,H}$ outside the cluster ($\mathrm{R3_H}$):

$$k_{3,H}([\mathrm{S6K^P}]) = c_3 \left(1 + \xi_3 \cdot \frac{[\mathrm{S6K^P}]^{\mathrm{n_3}}}{K_3^{n_3} + [\mathrm{S6K^P}]^{\mathrm{n_3}}}\right),$$

and $k_{3,C}$ changed to $k_{3,C,H}$ ($\mathrm{R3_{C,H}}$):

$$k_{3,C,H}(x) = c_3 \left(1 + \xi_3 \cdot \frac{[\mathrm{S6K^P}]^{\mathrm{n_3}}}{K_3^{n_3} + [\mathrm{S6K^P}]^{\mathrm{n_3}}}\right).$$

The corresponding parameter vector with the extra two parameters, n_3 and K_3, is:

$$\theta = (c_1, c_{-1}, c_2, c_{-2}, K_{-2}, c_3, \xi_3, n_3, K_3, c_4, c_5, c_6, c_7, \xi_6, c_8, c_{-8}, Q_{\mathrm{wt}},\\ s6k_0, \xi_{[\mathrm{S6K_c}]_0}, si8_{\mathrm{effect}}, si9_{\mathrm{effect}}, s_1, s_2, s_3, s_4, s_5, \sigma_1, \sigma_2, \sigma_3, \sigma_4, \sigma_5)^\top.$$

1.6 Extended Model with Clustering (non-l-dependent)

In the main manuscript, we only discuss in detail the models with clustering where the clustering rate is l dependent, *Extended Model with Clustering* and *Extended Model with Clustering and Nonlinear Translation.* To show the effect of l dependence, we also explore the models without clustering, where the rate is not l dependent-*Extended Model with Clustering* (non-l-dependent) and *Extended Model with Clustering and Nonlinear Translation* (non-l-dependent). We compare the four models to demonstrate why l dependence might be necessary to explain all data observations in Sec. 9.1.

The *Extended Model with Clustering* (non-l-dependent) shares the same state vector, initial condition, ODE system, and the parameter vector of the *Extended Model with Clustering (l-dependent)*, with only k_8 changed to $k_8(l) = c_8$.

1.7 Extended Model with Clustering and Nonlinear Translation Rate (non-l-dependent)

The *Extended Model with Clustering and Nonlinear Translation* (non-l-dependent) shares the same state vector, initial condition, ODE system, and parameter vector of the *Extended Model with Clustering and Nonlinear Translation* (l-dependent), with only k_8 changed to $k_8(l) = c_8$.

2 Activation function, l, as a function of CAG repeat length q

To model how CAG repeat length (q) affects the binding affinity function k_1 and clustering rate function k_8, we use hairpin length (Fig. 1C), constructed to be a function of q, as part of k_1 and k_8 functions. We use hairpin length, rather than q directly, because MID1 binds with RNA through the stable hairpin structure. Without the hairpin structure, which can happen when q is very small, the binding affinity with MID1 and thus the clustering rate might be very weak or non-existent.

Based on the in silico RNA-structure study of CAG repeat stretches with different repeat sizes,[1] hairpin length changes very little when q is small, while the hairpin length linearly increases after a certain q threshold (Fig. 1A). A linear function of q can not reproduce such dynamics. We thus model hairpin length in the form of an "activation function":

$$\log(1 + \exp(a \cdot (q - b))) + c.$$

However, notice that the hairpin length is seven for both 17 CAG and 21 CAG repeat length. To reduce the number of parameters, we let $c = 7$. Choosing $(a = 1.45, b = 30)$ to be the initial guess of the optimization, the curve fitting result (Fig. 1B) is:

$$\log(1 + \exp(1.400236314427246 \cdot (q - 20.95079181127341))) + 7.$$

As the binding affinity or the clustering rate should possibly be non-existent when q is small, we offset the hairpin length function and define l to be:

$$l(q) = \log(1 + \exp(1.400236314427246 \cdot (q - 20.95079181127341))).$$

The binding affinity and clustering rate are thus in the form of:

$$k_1(l(q)) = c_1 \cdot l(q) = c_1 \cdot \log(1 + \exp(1.400236314427246 \cdot (q - 20.95079181127341))),$$
$$k_8(l(q)) = c_8 \cdot l(q) = c_8 \cdot \log(1 + \exp(1.400236314427246 \cdot (q - 20.95079181127341))).$$

3 Existence, uniqueness, and non-negative invariance of ODE solutions proof

Proposition. *Consider an ODE system*

$$\dot{x} = f(x), \quad x(0) = x_0 \in \mathbb{R}^{n_x}_{\geq 0},$$

with the following properties:

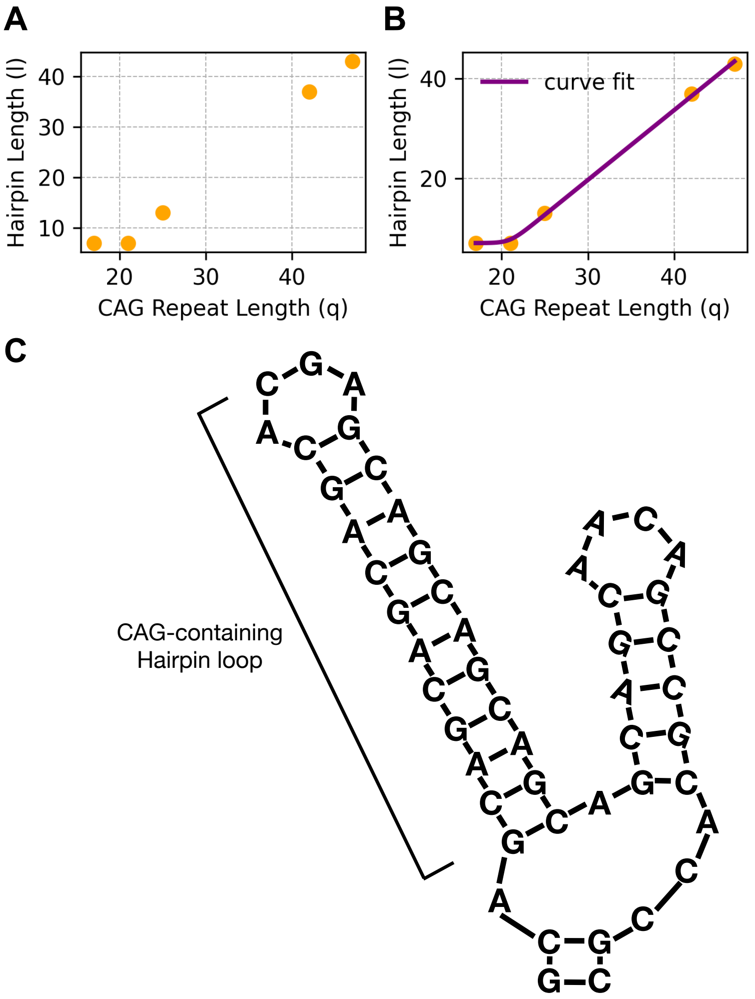

Figure 1: **Relationship between CAG repeat length and hairpin structure length.** (A) Hairpin length ($l(q)$) plotted against CAG repeat length (q), based on an in silico RNA secondary-structure prediction study.[1] The hairpin length shows minimal change at low CAG repeat numbers but increases linearly beyond a threshold repeat length. (B) Curve fitting with an "activation function" to capture the nonlinear relationship between CAG repeat length and hairpin length. (C) Representative illustration of a CAG-containing RNA hairpin structure, highlighting the formation of stable secondary structures responsible for MID1 binding affinity and RNA clustering.

(A1)* *Regularity. *f is C^1 on an open set containing $\mathbb{R}^{n_x}_{\geq 0}$*

(A2)* *Inward boundary. *For each i, $f_i(x) \geq 0$ whenever $x_i = 0$ and $x \in \mathbb{R}^{n_x}_{\geq 0}$*

(A3)* *Uniform bounds. *There exist nonnegative linear functionals $T_k(x) = \alpha_k \cdot x$ and constants $a_k \geq 0$, $b_k > 0$ such that*

$$\dot{T}_k(x) \;\leq\; a_k - b_k T_k(x),$$

and the T_k collectively bound all coordinates.

Then the initial value problem admits a unique global solution $x : [0, \infty) \to \mathbb{R}^{n_x}_{\geq 0}$, and the nonnegative orthant is forward invariant.

Sketch of proof. By (A1), f is locally Lipschitz, so there is a unique local solution (Picard–Lindelöf). By (A2), the vector field points inward at each boundary face, hence solutions starting nonnegative remain nonnegative. By (A3), Grönwall's inequality implies uniform bounds on each T_k, and thus on all components of $x(t)$. The trajectory remains in a compact, positively invariant set on which f is globally Lipschitz. Therefore, local solutions extend uniquely for all $t \geq 0$. □

Remark. *In the models in this work, assumptions (A1)–(A3) are satisfied as follows:*

- *Regularity (A1) holds since all right-hand sides consist of polynomials or smooth rational/Hill-type functions with strictly positive denominators.*
- *Inward boundary condition (A2) is guaranteed because all degradation or loss terms are proportional to the state variable itself and vanish at the boundary, while formation terms remain non-negative.*
- *Uniform bounds (A3) are obtained from biologically meaningful totals (e.g. conserved pools of S6K, MID1, or RNA) and clearance terms that yield differential inequalities of the form $\dot{T} \leq a - bT$.*

Therefore, each specific model variant is a special case of the proposition.

4 Experimental Condition

The dataset[1] comprises 14 experiments, of which 11 are *in vitro* and 3 are *in vivo*. Each experiment is characterized by six binary or numerical variables, which we denote as experimental conditions $u^{(c_e)}$, $c_e \in \{1, 2, \ldots, 6\}$ and collect into a vector as input $u_i = \{u_i^{(c_e)}\}_{c_e=1}^{6}$, $i \in \{1, 2, \ldots, 14\}$. The six experimental conditions are: (1) $u^{(1)} = allele_1$: whether allele 1 expresses (binary), (2) $u^{(2)} = allele_2$: whether allele 2 expresses (binary), (3) $u^{(3)} = q_1$: the CAG repeat length of allele 1 (non-negative), (4) $u^{(4)} = q_2$: the CAG repeat length of allele 2 (non-negative), (5) $u^{(5)} = si8_{\text{flag}}$: whether siRNA si-8 is applied (binary), (6) $u^{(6)} = si9_{\text{flag}}$: whether siRNA si-9 is applied (binary). Note that if $u^{(1)} = 0$ ($u^{(2)} = 0$), then $u^{(3)} = 0$ ($u^{(4)} = 0$) as the corresponding allele is not expressed.

The experimental conditions influence the dynamics in three main ways: allele expression, CAG repeat length, and MID1 knockdown. First, if an allele is expressed, transcription for

that allele is “on,” with the rate implemented as $k_5 = c_5 \cdot u^{(\zeta)} = c_5 \cdot allele_\zeta$. In the *Baseline Model*, where RNA levels are constant, the initial RNA level is set to $[\text{RNA}]_0 = rna_0 \cdot u^{(\zeta)} = rna_0 \cdot allele_\zeta$. For a non-expressing allele, this value is zero. Second, the values q_1 and q_2 ($u^{(3)}$ and $u^{(4)}$) enter the system through the l function (see Sec. 2) Third, the Application of siRNA si-8 or si-9 reduces MID1 levels. This is modeled by setting the initial condition for MID1 as $[\text{MID1}]_0 = \exp(si8_{\text{effect}} \cdot u^{(5)} + si9_{\text{effect}} \cdot u^{(6)}) = \exp(si8_{\text{effect}} \cdot si8_{\text{flag}} + si9_{\text{effect}} \cdot si9_{\text{flag}})$. The specific values of these six experimental conditions for each experiment are listed in Table 1.

Table 1: **Experimental conditions.** Experimental configurations of data,[1] showing experimental conditions $u^{(c_e)}$, $c_e \in \{1, 2, \ldots, 6\}$ in each experiment.

	**Experiment**	$u^{(1)}$	$u^{(2)}$	$u^{(3)}$	$u^{(4)}$	$u^{(5)}$	$u^{(6)}$
In Vitro	1: q17	1	0	17	0	0	0
	2: q49	0	1	0	49	0	0
	3: q20 control	1	0	20	0	0	0
	4: q20 MID1 si-8	1	0	20	0	1	0
	5: q20 MID1 si-9	1	0	20	0	0	1
	6: q51 control	0	1	0	51	0	0
	7: q51 MID1 si-8	0	1	0	51	1	0
	8: q51 MID1 si-9	0	1	0	51	0	1
	9: q83 control	0	1	0	83	0	0
	10: q83 MID1 si-8	0	1	0	83	1	0
	11: q83 MID1 si-9	0	1	0	83	0	1
In Vivo	12: wt/wt	1	1	Q_{wt}	Q_{wt}	0	0
	13: wt/q111	1	1	Q_{wt}	111	0	0
	14: q111/q111	1	1	111	111	0	0

5 Optimized Q_{wt} distribution

In the experimental conditions wt/wt and wt/Q111, the CAG repeat length of the wild-type allele is not experimentally determined. We therefore treat this quantity, denoted Q_{wt}, as a

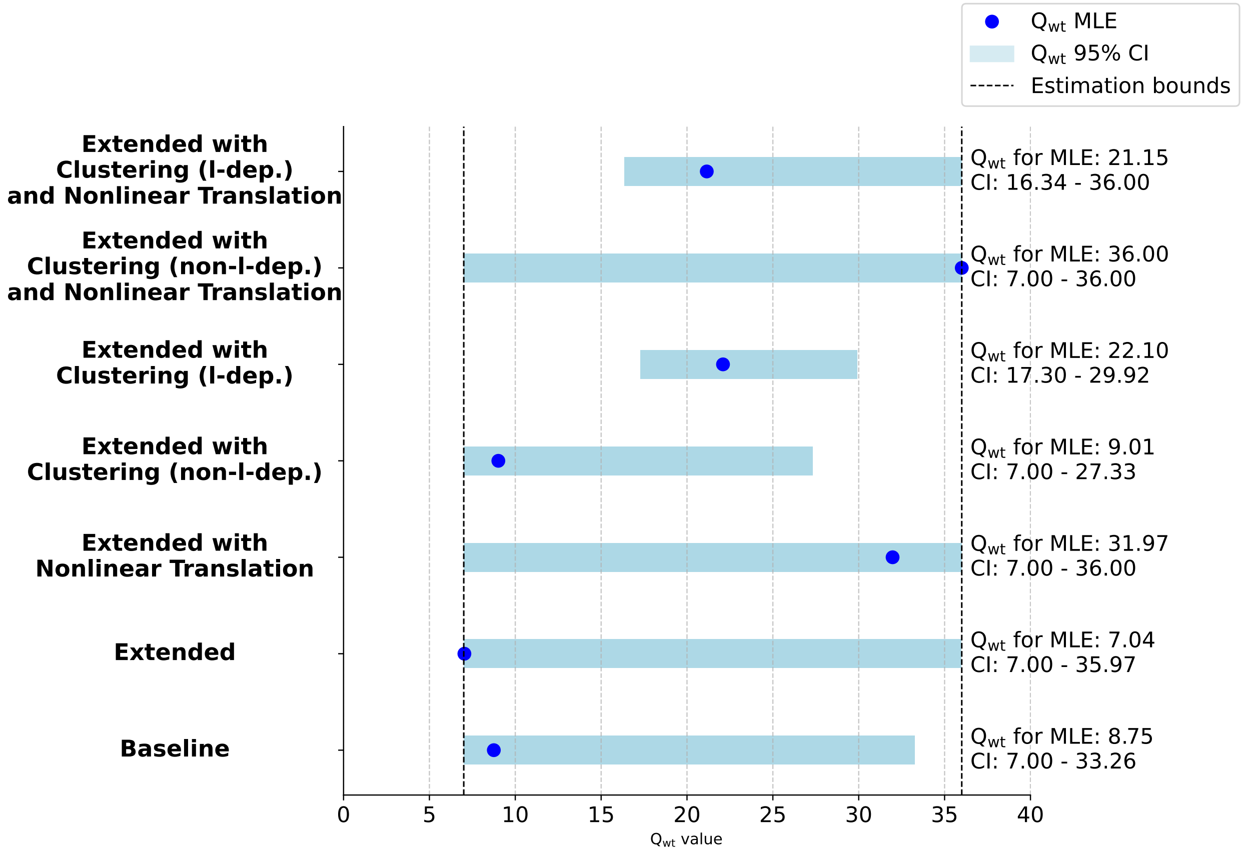

Figure 2: **Estimated Q_{wt} across models.** Blue dots indicate the maximum likelihood estimate (MLE) of Q_{wt}; black dashed lines mark the estimation bounds. Black error bars represent 95% confidence intervals, calculated from optimization endpoint distributions.

free parameter to be estimated during model calibration. Fig. 2 summarizes the maximum likelihood estimate (MLE) and 95% confidence intervals (CI) of Q_{wt} for all model variants. CIs were obtained using parameter values of the endpoints of model optimization that had a final value of the objective function below the CI threshold.

For simplicity in estimation, we treat Q_{wt} as a continuous rather than a discrete quantity, even though CAG repeat numbers are inherently integer valued. This choice has a negligible impact on model behavior, as the hairpin length function $l(q)$ used in all model variants is continuous in q, ensuring that small fractional differences in Q_{wt} produce smooth and well-defined effects on downstream dynamics.

The 95% confidence intervals indicate a high uncertainty of the parameter estimate for most models, some even covering the whole estimation space. However, the uncertainty is reduced for the two models with the best objective function values – the *Extended Model with Clustering (l-dep.) and Nonlinear Translation* and the *Extended Model with Clustering (l-dep.)*. An interesting thing to point out is that the MLE, the best parameter value obtained, for

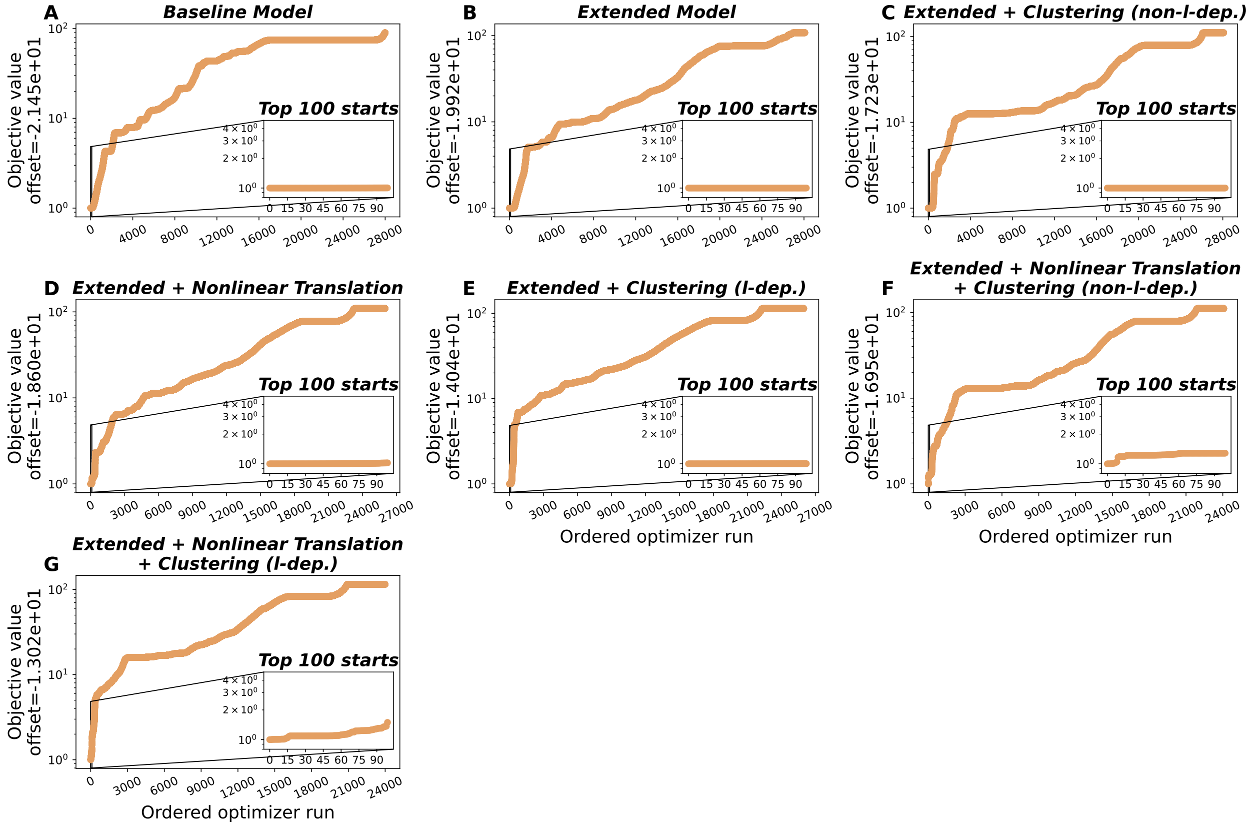

Figure 3: **Waterfall plots for all seven models considered in this study.** Each curve shows the negative log-likelihood (NLLH) values from independent optimization runs, sorted from best to worst. The y-axis is shown in log scale and offset by the best fit value, such that the minimum appears at 1; the applied offsets are indicated in the y-axis labels. Insets display the top 100 runs, with the y-axis scaled to the 95% confidence region (corresponding to an NLLH increase of $\chi^2_{0.95,df=1}$ from the best fit). Rapid plateauing near the minimum indicates frequent convergence to the same optimum.

these models is just above 20 repeats, from ∼21 to ∼23.

6 Convergence of optimization

To assess convergence in parameter estimation, we generated waterfall plots for all models considered (Fig. 3). Each plot shows the Negative Log-likelihood (NLLH) values obtained from independent optimization runs, sorted from best to worst.

Ideally, for well-constrained models with sufficient data, the objective function values from the best-performing optimization starts would rapidly plateau, indicating that the optimizer frequently converges to the same—or nearly the same—minimum. For more complicated

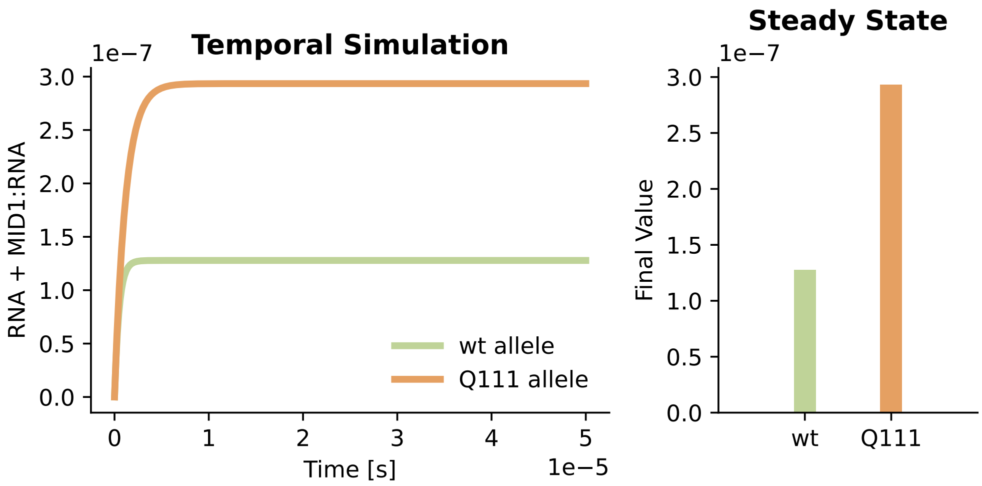

Figure 4: **Comparison of total RNA + MID1:RNA levels for wt and Q111 alleles in the *Extended Model.*** Left: Time-course simulation showing total RNA (free and MID1-bound) for wt (green) and Q111 (orange) alleles under identical transcription rates. Right: Corresponding steady-state values, indicating consistently higher RNA abundance for Q111 allele. This imbalance arises from the lower degradation rate of MID1-bound RNA (c_7) relative to free RNA (c_6), leading to preferential stabilization of Q111 RNA.

models, the top ∼100 starts do not always show just one plateau. Given the size and complexity of these models, coupled with the relatively limited amount of available experimental data, small differences in objective values among the top fits likely arise from flat directions in parameter space. In such cases, multiple parameter combinations can yield similarly good fits, but with slight numerical variation due to the optimizer exploring these nearly equivalent regions.

7 Steady-state RNA and MID1:RNA level comparison between wt and Q111 alleles

We examined the steady-state total RNA levels (sum of free and MID1-bound species) for both alleles in the *Extended Model.* As shown in Fig. 4, the Q111 allele consistently reaches a higher translatable resource abundance than the wt allele under identical transcription rates. This result provides a mechanistic explanation for the competitive expression pattern observed *in vivo*: the lower degradation rate of MID1-bound RNA (c_7) compared to free RNA (c_6) leads to preferential stabilization of Q111 RNA, which binds MID1 more strongly. The temporal simulation (left panel) shows this difference emerging over time, while the bar plot (right panel) summarizes the steady-state values, highlighting the persistent imbalance.

8 Parameter identifiability analysis

To assess parameter identifiability, we computed profile likelihoods for all mechanistic parameters across all models. The profile likelihood for parameter θ_m is defined as

$$\mathrm{PL}_m(p) = \max_{\theta \in \{\theta \,|\, \theta_m = p\}} \mathcal{L}_{\mathcal{D}}(\theta),$$

and the corresponding confidence interval is given by

$$\mathrm{CI}^{\alpha}_{\theta_m} = \left\{ \theta_m \,\middle|\, \frac{\mathrm{PL}_m(\theta_m)}{\mathcal{L}_{\mathcal{D}}(\hat{\theta})} \geq \exp\left(-\frac{\Delta^1_\alpha}{2}\right) \right\},$$

where Δ^1_α is the α-th percentile of the χ^2_1 distribution. A parameter is structurally non-identifiable if its profile likelihood is flat across the entire estimation range, indicating that the parameter value has no influence on the model output given the available observables, regardless of the amount of data. A parameter is practically non-identifiable if its confidence interval extends to one or both parameter bounds, indicating that the available data is insufficient to constrain the parameter within the estimation range. To improve robustness of the computed profiles, we performed profiling from multiple optimization starting points and retained the maximum profile ratio at each grid point.

Profile likelihoods for all seven models are shown in Figs. 5–11. Across all models, the majority of kinetic parameters are non-identifiable. While the dataset comprises 48 measurements, these correspond to only 14 distinct experimental conditions, with replicates within each condition providing information about measurement noise but not additional constraints on mechanistic model parameters. Constraining 23–30 parameters from 14 independent conditions leads to widespread non-identifiability, as expected. Specifically, as all the available data is obtained at steady state, the forward and backward rates of reversible reactions can compensate for each other to produce the same steady state. For instance, the S6K phosphorylation parameters (c_2, c_{-2}) are consistently non-identifiable across all models. Moreover, as the only observed quantity in the observational model is the downstream protein concentration, the basal translation rate c_3 and transcription rate c_5 are non-identifiable in all models.

Several parameters are, however, better constrained. The wild-type CAG repeat length Q_{wt} is practically identifiable in l-dependent clustering models (see also Sec. 5). The siRNA knockdown effect $si9_{\text{effect}}$ is practically identifiable in most models, as multiple experimental conditions with and without MID1 knockdown are available in the data, while $si8_{\text{effect}}$ is practically identifiable in only one of seven models. In the *Extended Model*, the individual degradation rate constants c_6 and c_7 are non-identifiable, spanning most of the estimation range. However, their ratio $c_6/c_7 \approx 2.5$ is maintained consistently across the confidence region as shown in Fig. 4B of the main text.

For non-identifiable parameters, the maximum likelihood estimate may appear at or near a parameter bound, reflecting the flatness of the objective function rather than restrictive bounds. Given that the estimation ranges already span up to 14 orders of magnitude for most kinetic parameters, extending the bounds beyond biologically plausible ranges is not justified. Moreover, for parameters whose non-identifiability is structural, extending bounds cannot resolve it. For ξ_3, which is constrained to $[1, 100]$ based on the assumption that the MID1-mediated enhancement of translation cannot be too large compared to the basal translation rate, the profile is flat or nearly flat across most of the range in most models, and varying the upper bound does not appreciably improve the model fit.

To examine parameter dependencies, we computed Spearman correlation matrices from the endpoints of multi-start optimization for all models (Figs. 12–18). Across models, consistent correlation patterns emerge. The MID1 binding and unbinding rates (c_1, c_{-1}) are strongly positively correlated, reflecting a trade-off between association and dissociation that preserves the effective binding equilibrium. Similarly, the S6K phosphorylation parameters (c_2, c_{-2}, K_{-2}) form a correlated block, indicating that the data constrains the net phosphorylation behavior but not the individual rate constants. In the *Extended Model*, c_5, c_6, and c_7 are positively correlated, as higher transcription rates can be compensated for by higher degradation rates to produce similar steady-state RNA levels. These correlation patterns are consistent with the non-identifiability observed in the profile likelihoods, as parameters within the same biochemical sub-process tend to co-vary while producing equal model outputs.

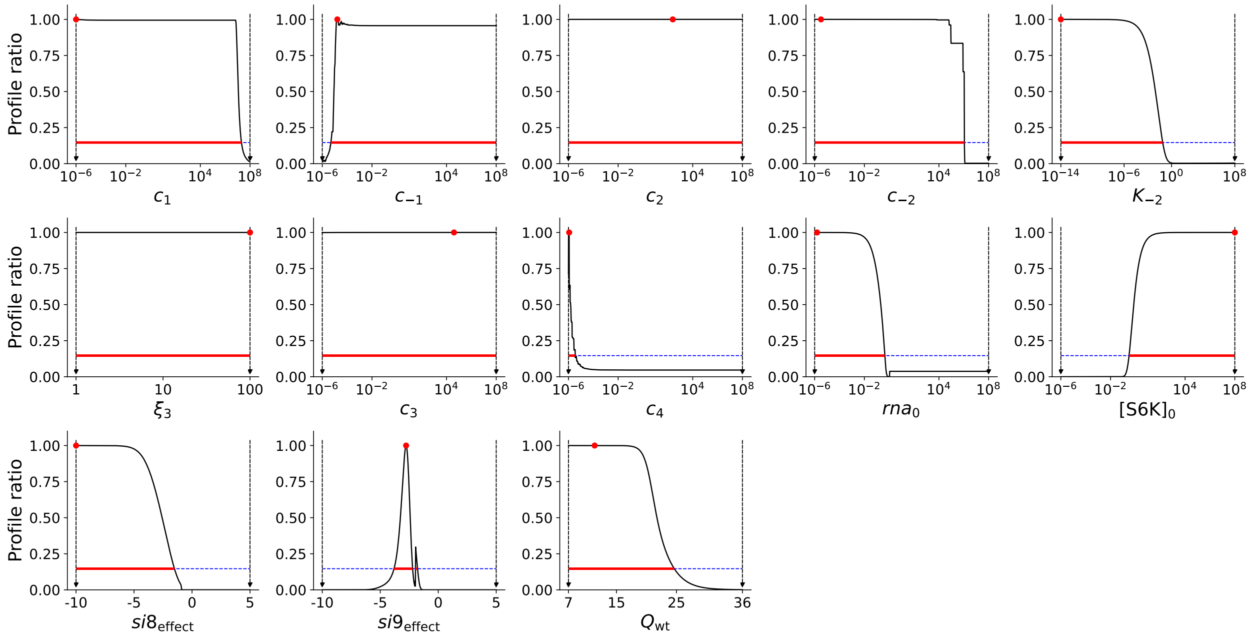

Figure 5: **Profile likelihoods for the *Baseline Model*.** Each panel shows the profile ratio as a function of one parameter. The red line indicates the 95% confidence threshold. Maximum likelihood estimates are indicated with red dots.

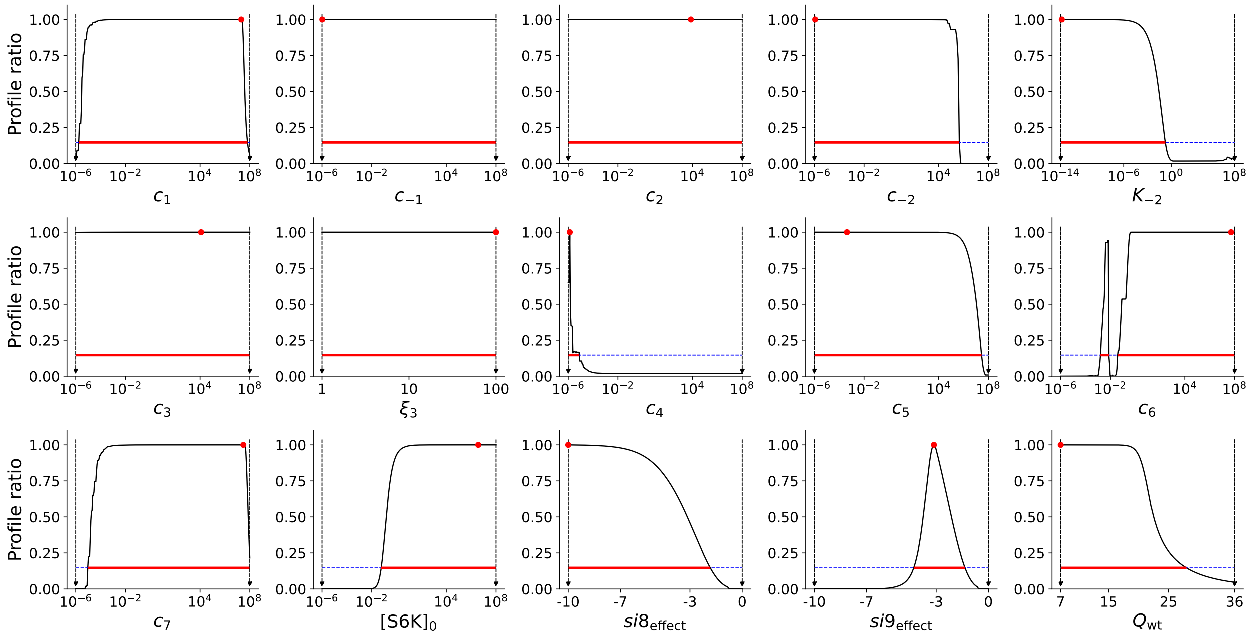

Figure 6: **Profile likelihoods for the *Extended Model*.**

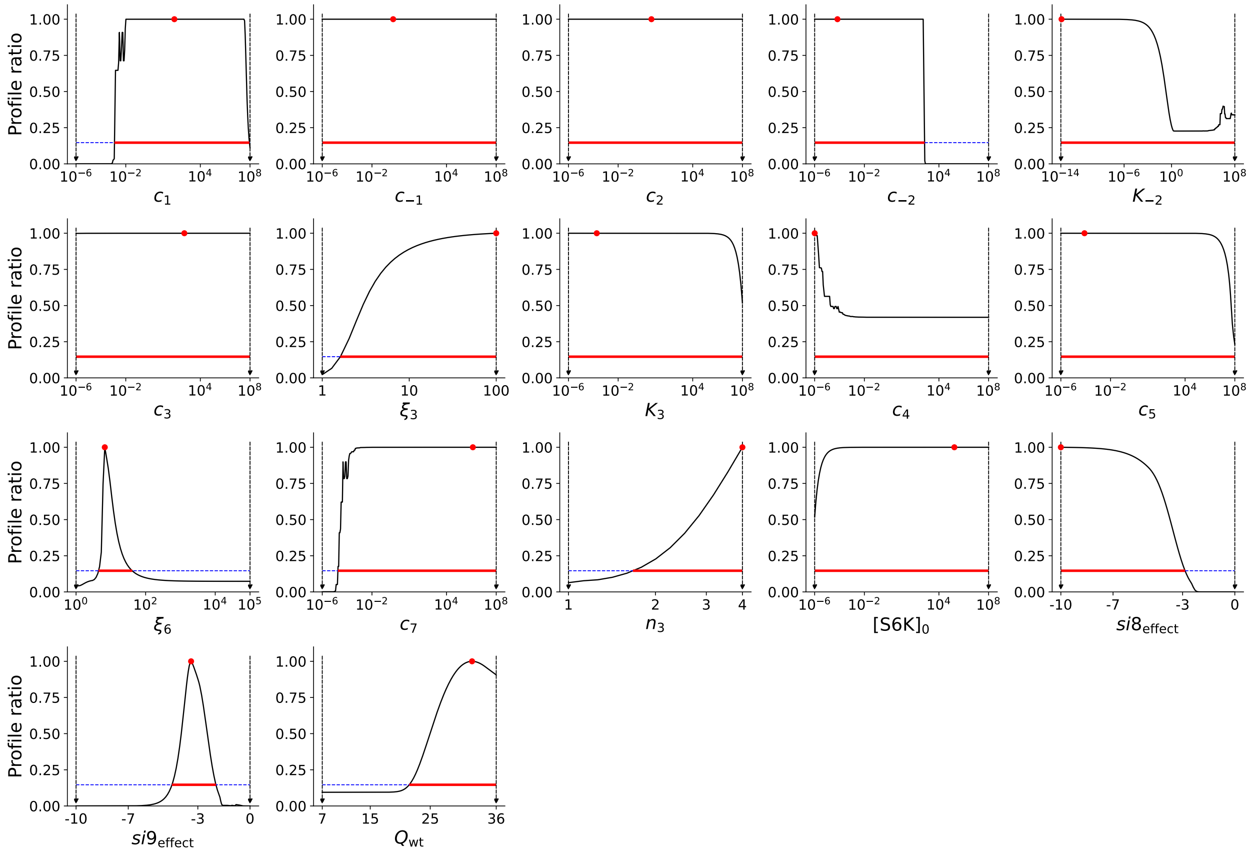

Figure 7: **Profile likelihoods for the *Extended Model with Nonlinear Translation*.**

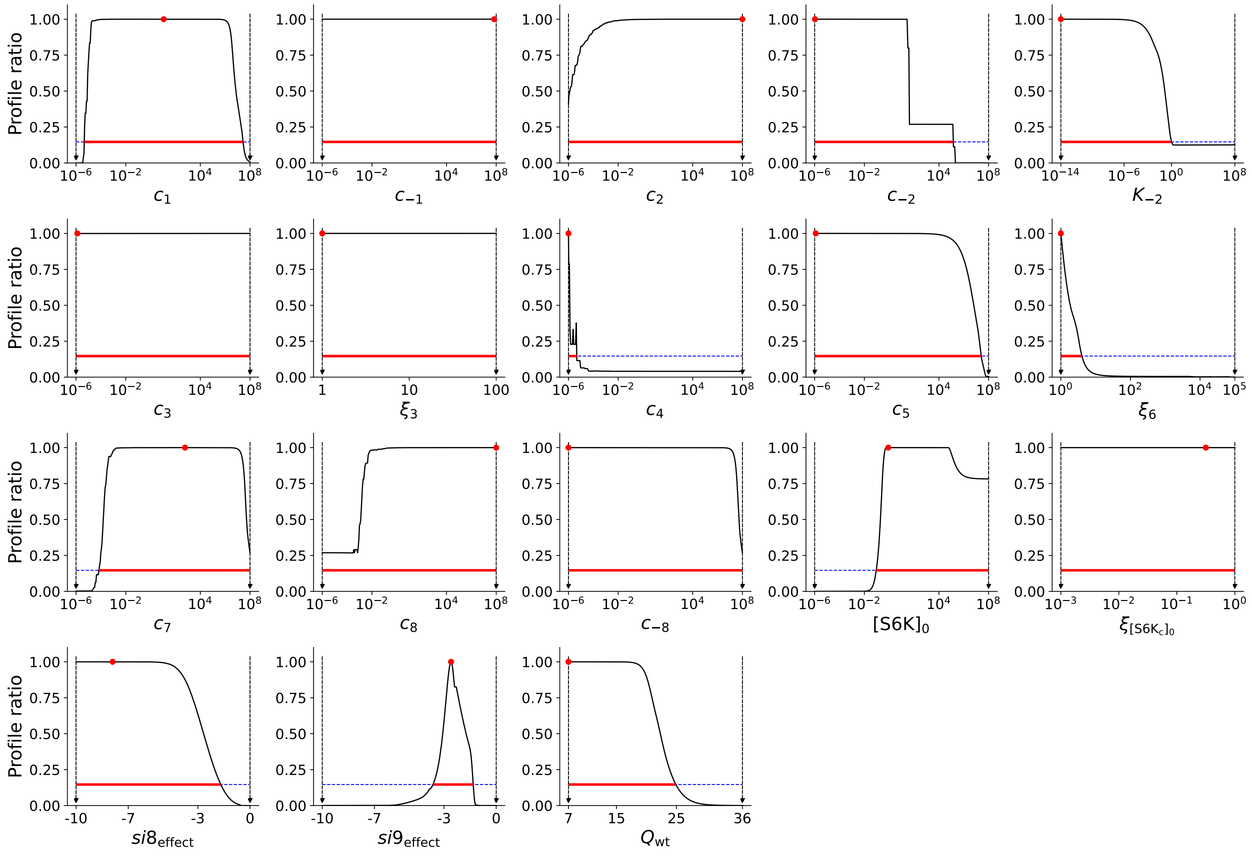

Figure 8: **Profile likelihoods for the *Extended Model with Clustering* (non-l-dependent).**

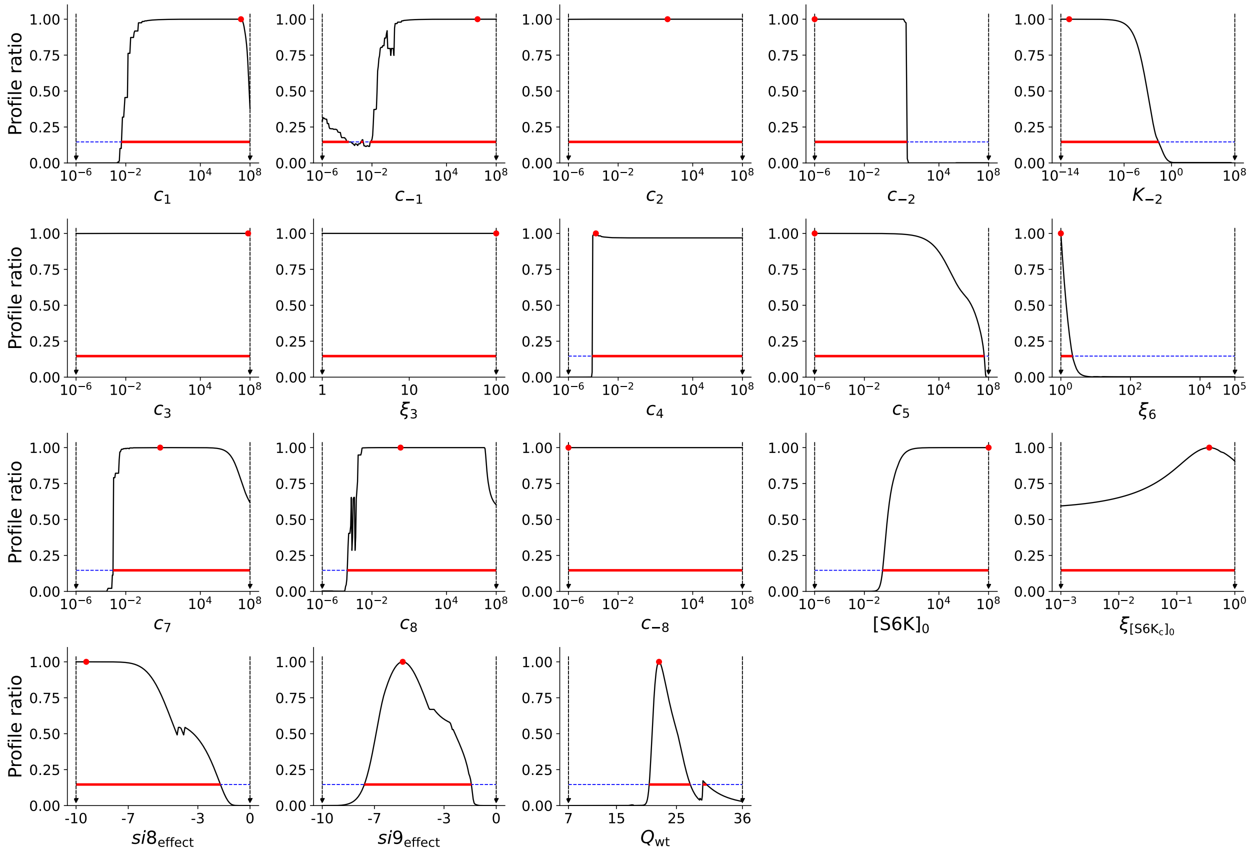

Figure 9: **Profile likelihoods for the *Extended Model with Clustering* (l-dependent).**

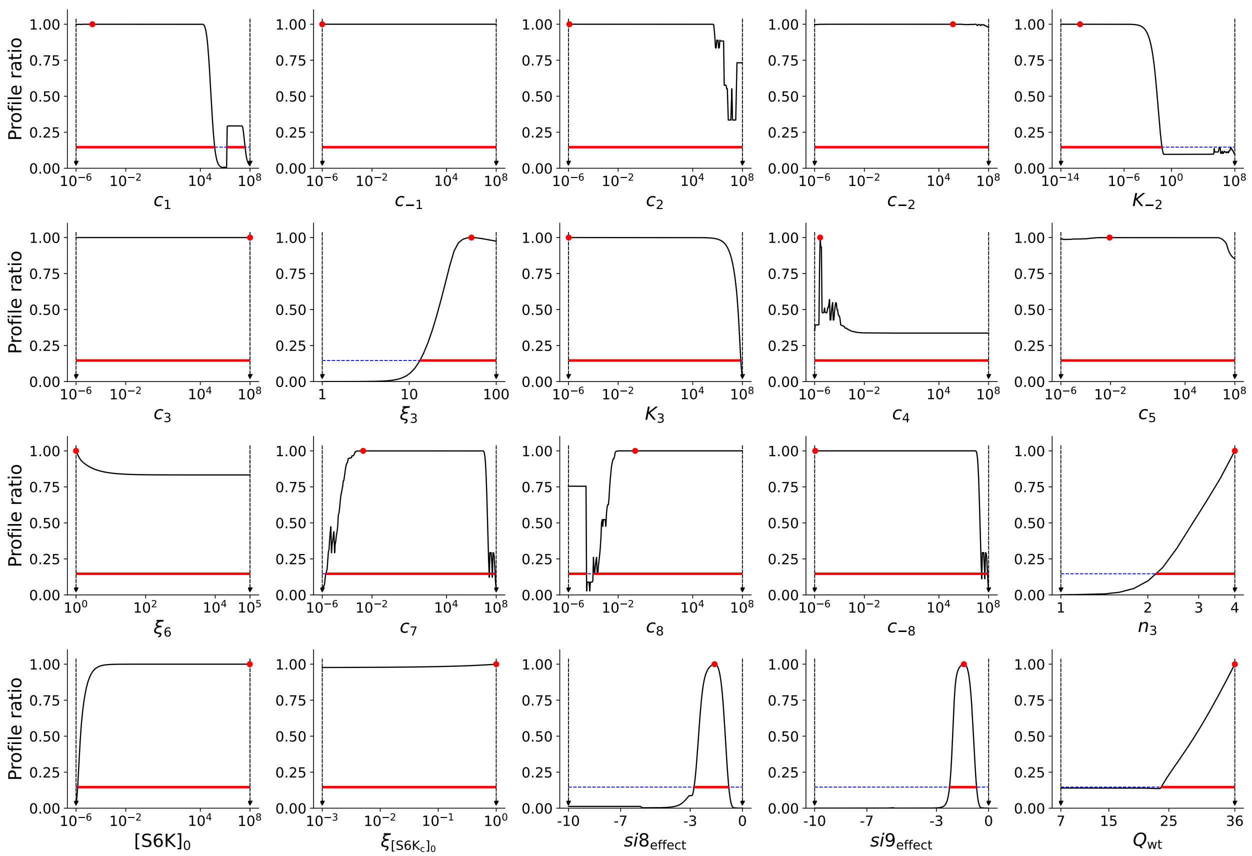

Figure 10: **Profile likelihoods for the *Extended Model with Clustering and Non-linear Translation* (non-l-dependent).**

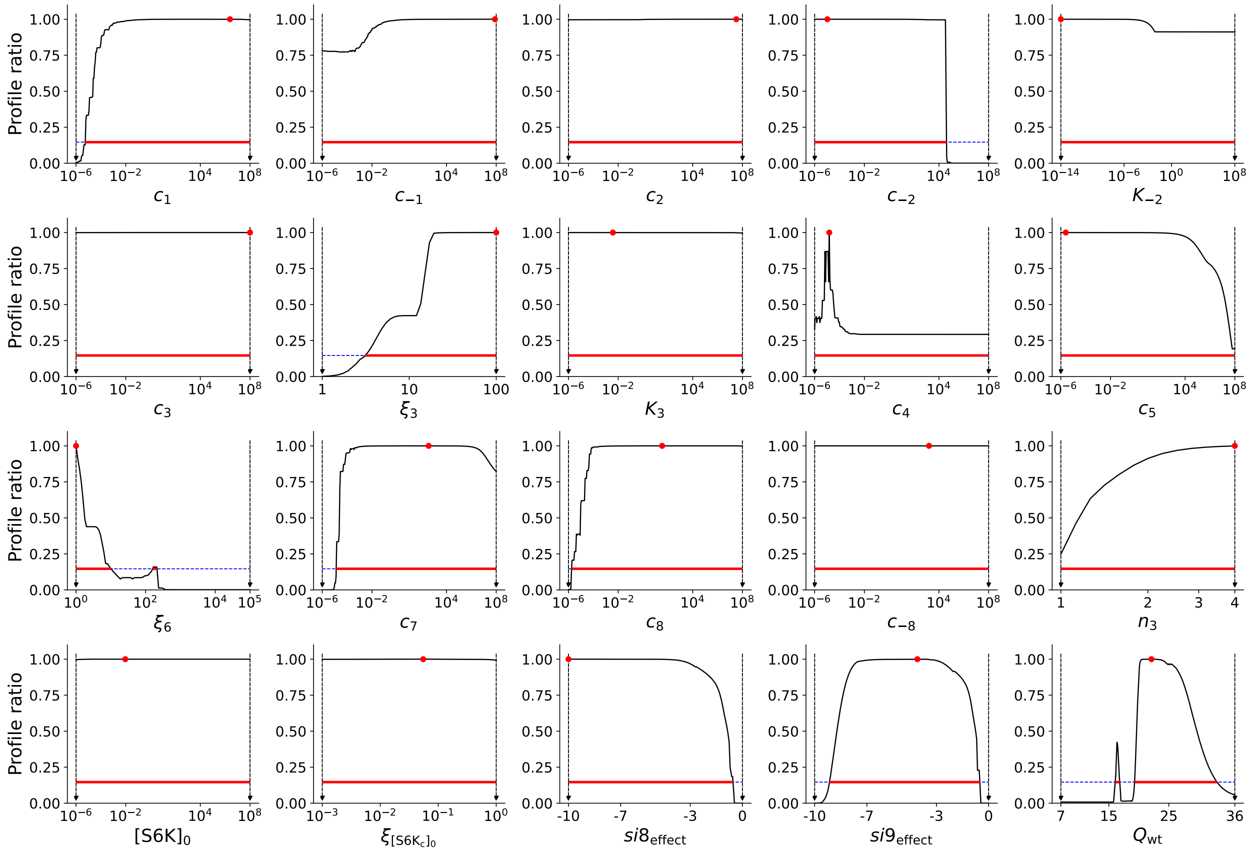

Figure 11: **Profile likelihoods for the *Extended Model with Clustering and Non-linear Translation* (l-dependent).**

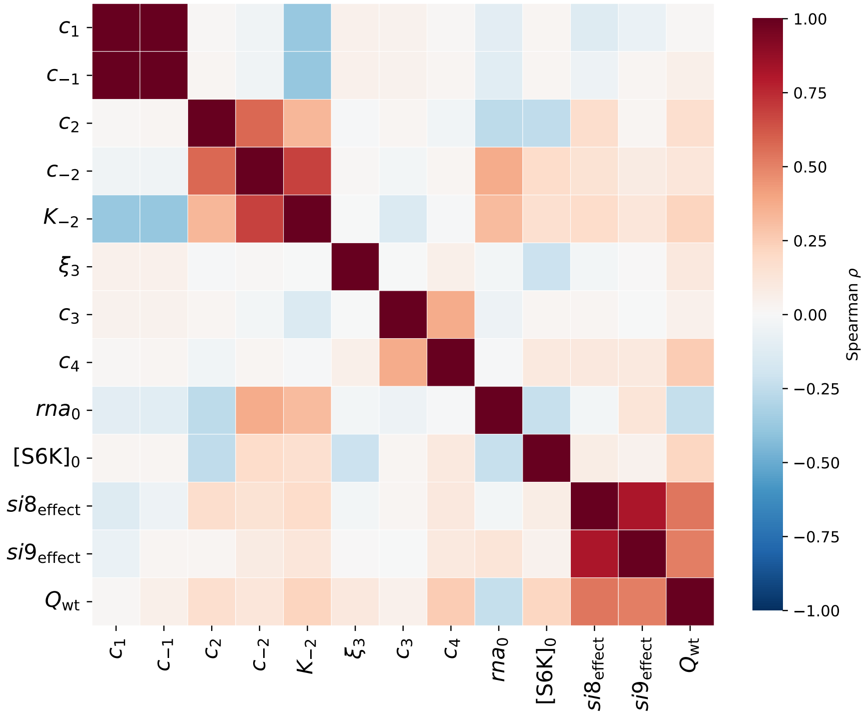

Figure 12: **Parameter correlation matrix for the *Baseline Model*.** Spearman correlation coefficients computed from multi-start optimization endpoints within the 95% confidence region.

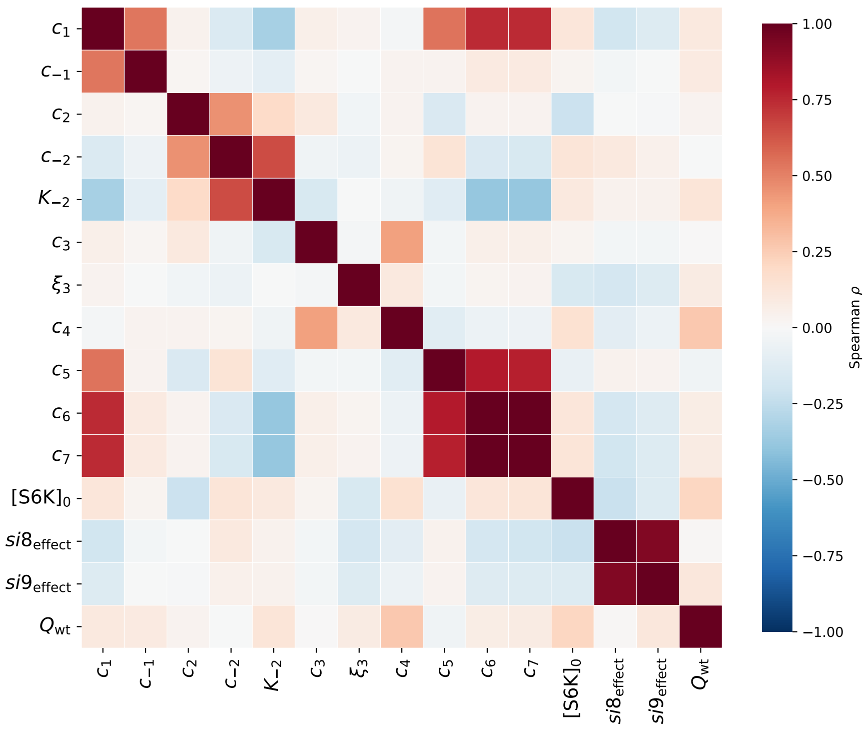

Figure 13: **Parameter correlation matrix for the *Extended Model*.**

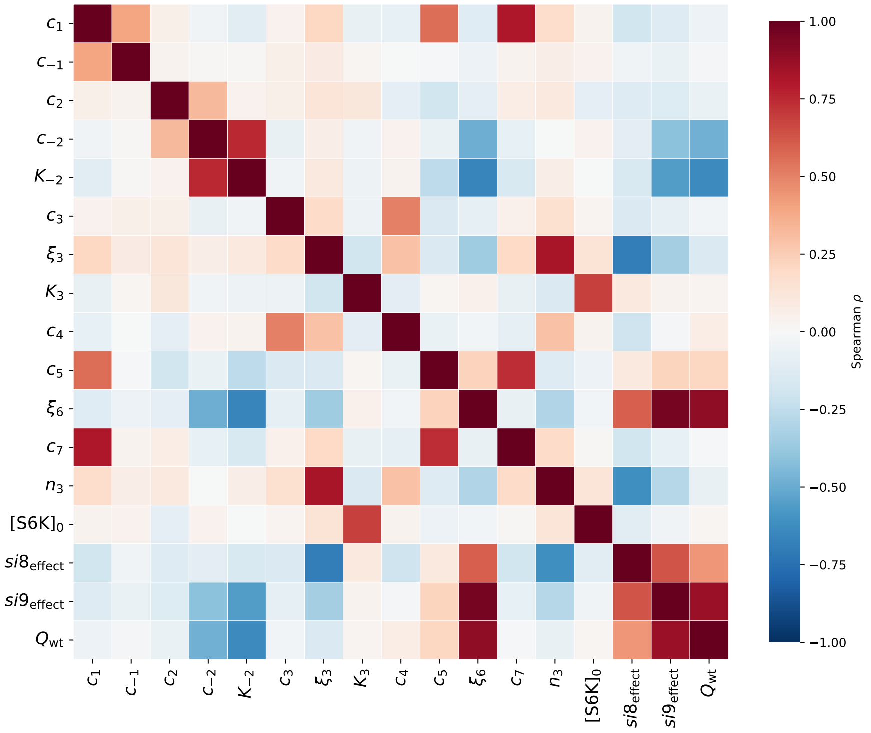

Figure 14: **Parameter correlation matrix for the *Extended Model with Nonlinear Translation*.**

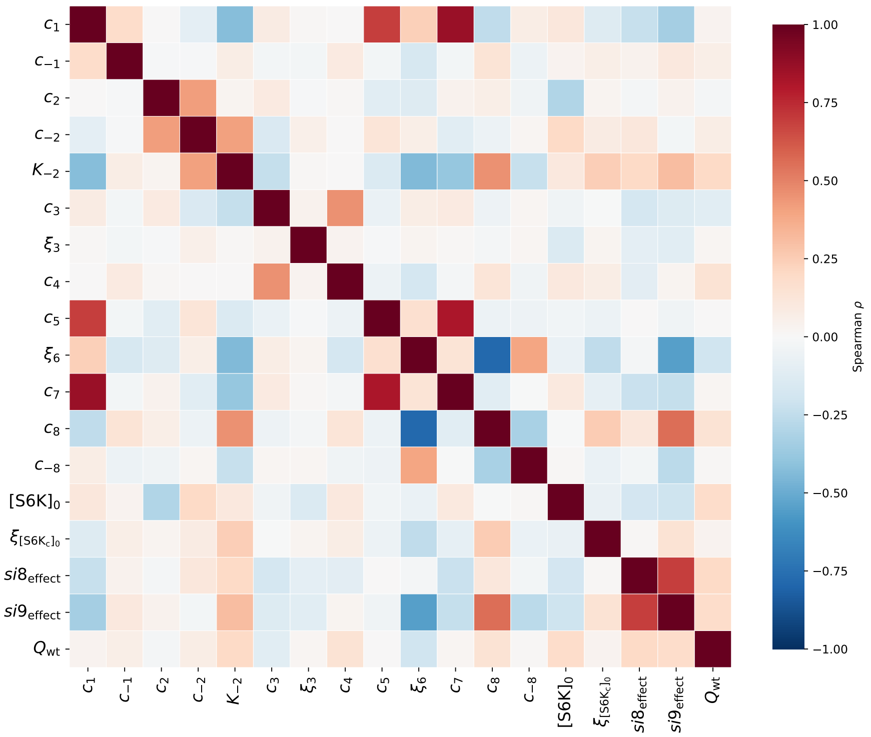

Figure 15: **Parameter correlation matrix for the *Extended Model with Clustering* (non-l-dependent).**

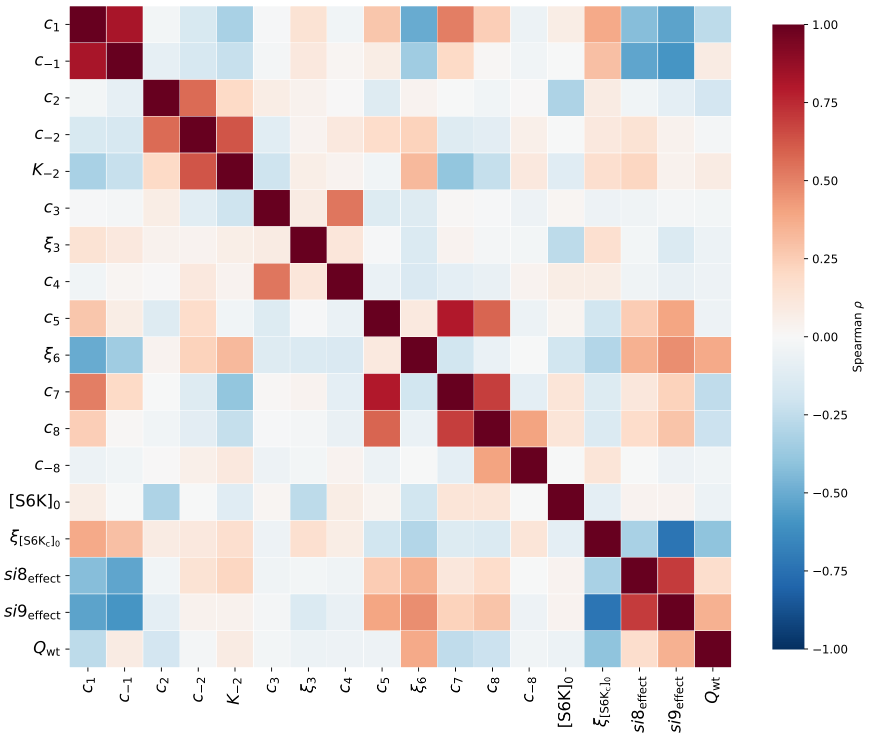

Figure 16: **Parameter correlation matrix for the *Extended Model with Clustering* (l-dependent).**

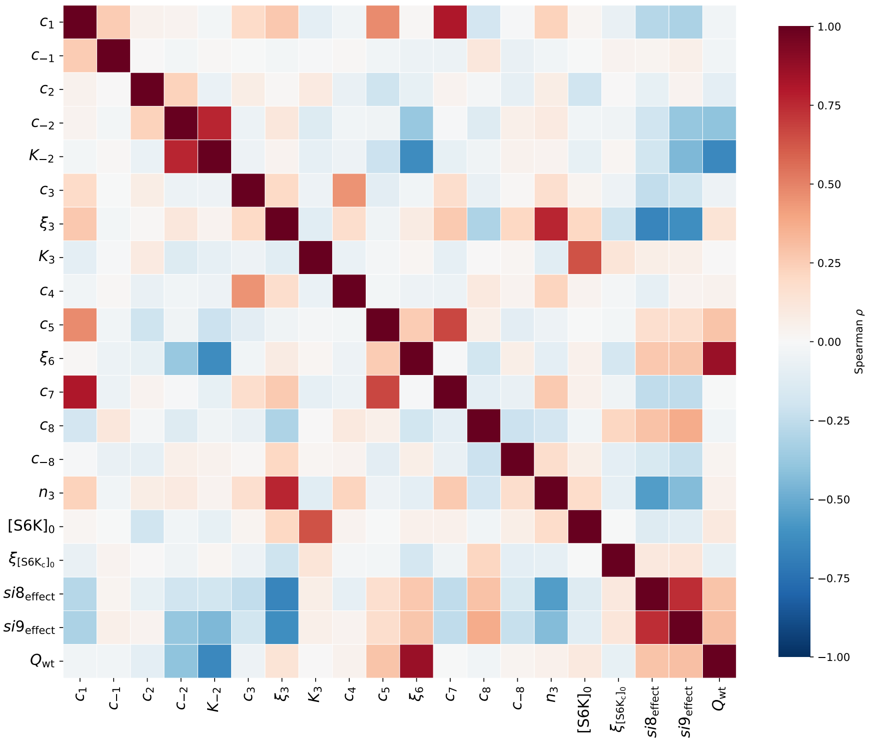

Figure 17: **Parameter correlation matrix for the *Extended Model with Clustering and Nonlinear Translation* (non-l-dependent).**

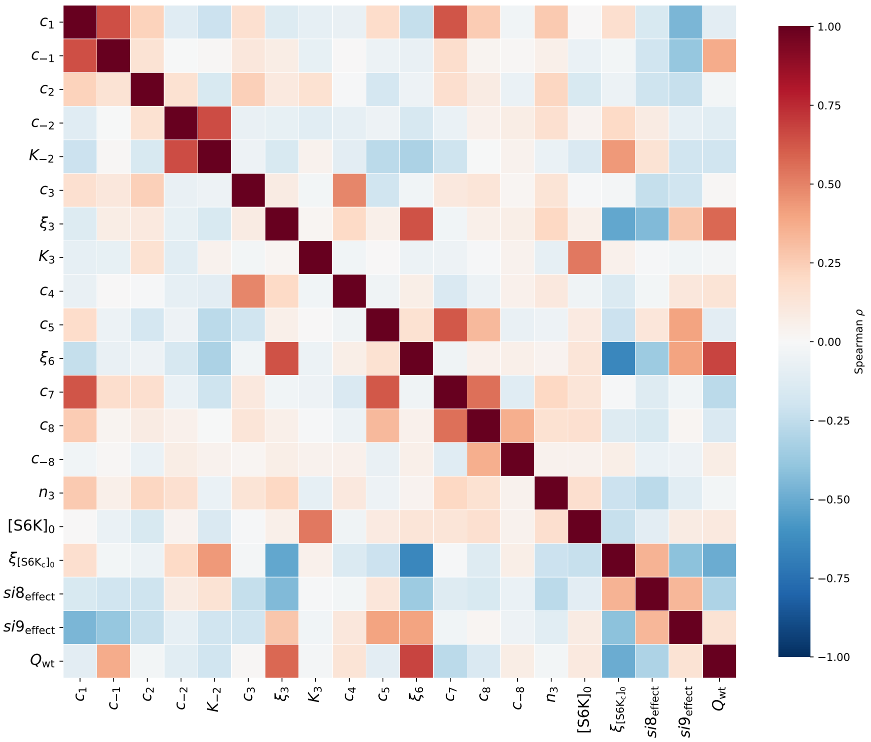

Figure 18: **Parameter correlation matrix for the *Extended Model with Clustering and Nonlinear Translation* (l-dependent).**

9 Comparison of fitting results of all models

To examine which model performs the best, we check the fitting results (Fig. 19), which are usually the most important indicator. Visual inspection of the fits reveals that the model incorporating both clustering and nonlinear translation—particularly the *Extended Model with Clustering and Nonlinear Translation* (l-dependent)—captures the observed patterns most accurately across all experimental conditions. In contrast, simpler models (e.g., the *Baseline Model*) misestimate HTT level in several conditions.

Model selection results using NLLH, AIC, AICc, and BIC (Table 2) provide a quantitative perspective on model performance. While the *Extended Model with Clustering and Nonlinear Translation* (l-dependent) achieves the lowest negative log-likelihood (NLLH = 14.02), indicating the best likelihood-based fit, the *Extended Model with Clustering* (l-dependent) without nonlinear translation achieves the best score in AIC. Other criteria, AICc and BIC, prefer simpler models, such as the *Baseline Model*. This discrepancy arises because NLLH reflects pure fit quality without penalizing complexity, while AIC, AICc, and BIC severely penalize the additional parameters introduced by nonlinear translation and clustering.

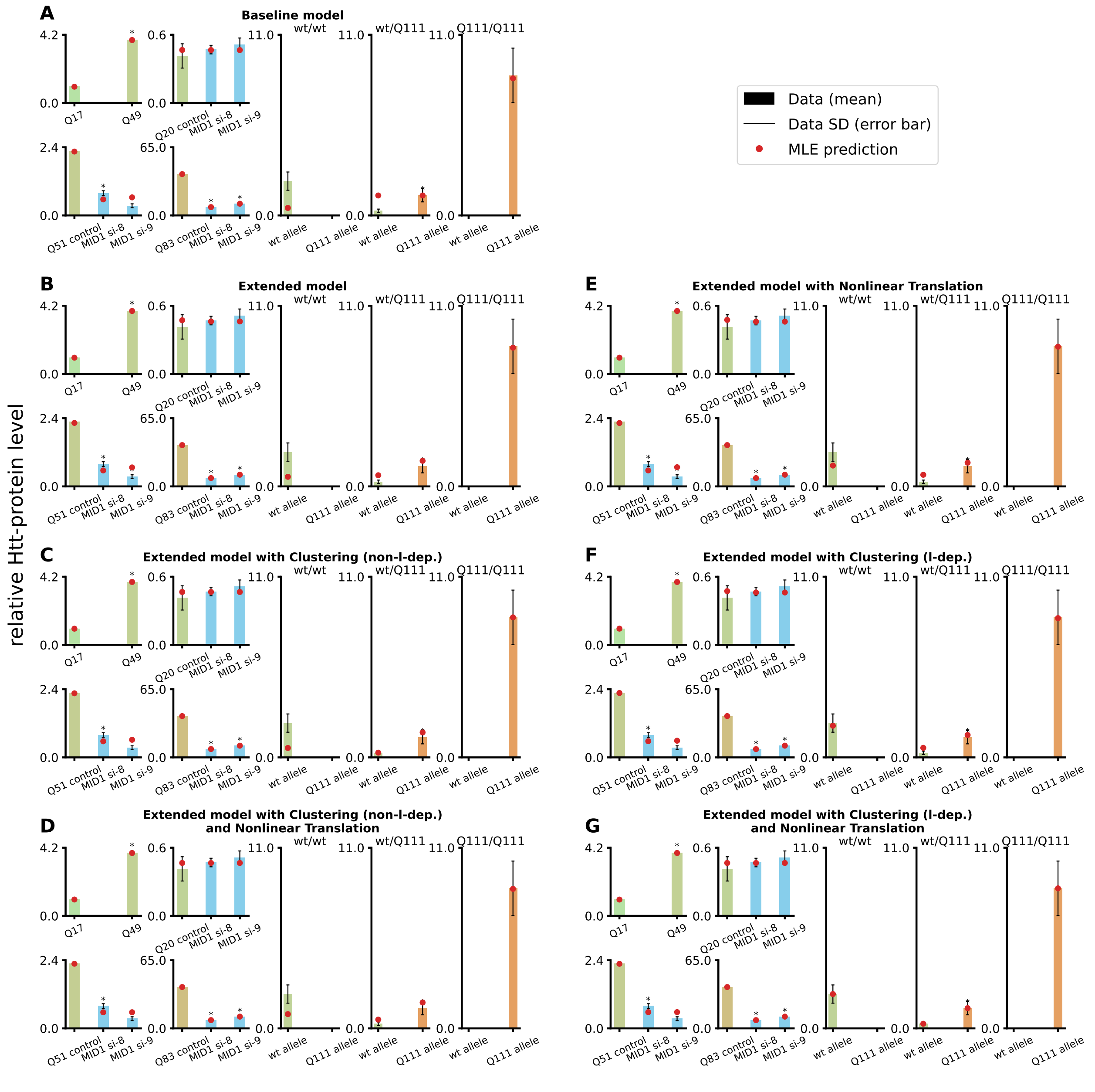

Figure 19: **Model fits for all models**. Bars indicate the mean relative HTT level (black line: standard deviation), with model predictions at maximum likelihood estimates (red dots). Panels compare the *Baseline Model*, *Extended Model*, *Extended Model with Nonlinear Translation*, *Extended Model with Clustering*, *Extended Model with Clustering and Nonlinear Translation*, and variants incorporating clustering without CAG repeat length dependence (non-l-dep.). Models with l-dependent clustering show improved agreement with the data, particularly in wt/wt and wt/Q111 settings.

Table 2: **Model comparison of all models using NLLH, AIC, AICc, and BIC.** Lower values indicate better performance. n_θ indicates the number of parameters. NT indicates Nonlinear Translation.

**Model**	**NLLH**	**AIC**	**AICc**	**BIC**	n_θ
Baseline Model	22.45	90.90	**136.90**	**133.94**	23
Extended Model	20.92	91.83	150.92	138.61	25
Extended + NT	18.23	90.47	166.07	140.99	27
Extended + Clustering (non-l-dep.)	19.60	95.20	180.68	147.60	28
Extended + Clustering (l-dep.)	15.04	**86.08**	171.55	138.47	28
Extended + Clustering + NT (non-l-dep.)	17.95	95.91	205.32	152.04	30
Extended + Clustering + NT (l-dep.)	**14.02**	88.04	197.45	144.18	30

9.1 l dependence is necessary for clustering rate

Interestingly, models without l-dependent clustering fail to or only barely capture the sublinear decrease in HTT level in wt/Q111 mice (Fig. 20). This failure to fit Observation 4 may indicate that CAG repeat length–dependent clustering is a necessary feature to reproduce the full spectrum of observed behaviors. Indeed, this differential clustering efficiency plays an important role in *Extended Model with Clustering and Nonlinear Translation* (l-dependent). As wt MID1-bound RNA is effectively excluded from the cluster compared to Q111 MID1-bound RNA in wt/Q111 mice, such RNA cannot collaboratively enhance the local S6K phosphorylation that occurs within the cluster, constraining the translation rate inside the cluster to not increase too much. Meanwhile, wt HTT level becomes sublinear in wt/Q111 setting due to wt MID1-bound RNA' restricted localization to the global compartment, where translation rate is at a lower basal rate. This basal translation level in wt/Q111 mice is lower compared to that observed in wt/wt mice, primarily because Q111 MID1-bound RNA is largely located inside the cluster.

The fact that the l-dependent clustering models compared to their respective variants without l-dependent clustering rank higher in both likelihood and penalized criteria highlights the importance of this mechanism in explaining the data. Models lacking l-dependence or clustering generally perform worse in all metrics, consistent with length-dependent clustering being an important component of the model.

Recent experimental work has reported CAG repeat length–dependent homotypic RNA clustering,[2] a phenomenon conceptually consistent with the clustering mechanism explored in our models. While those findings were observed within condensates, our framework suggests that analogous repeat-length–dependent clustering could arise in localized cytoplasmic translation hubs, where MID1-bound RNA may influence both spatial organization and local $S6K^P$–dependent translation. Such parallels offer an intriguing indication that length-

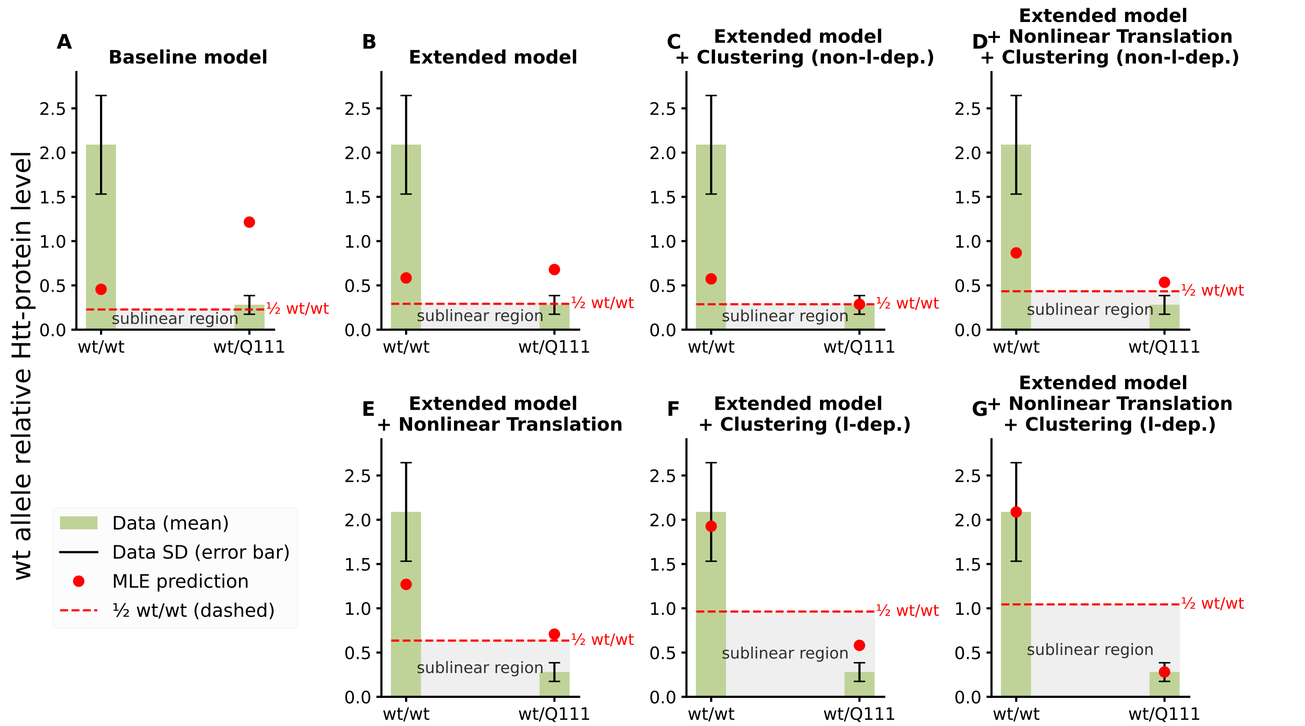

Figure 20: **Comparison of model predictions for wild-type allele–derived HTT levels in *wt/wt* and *wt/Q111* settings.** Green bars indicate experimental mean values with standard deviation (black error bars), red dots represent model predictions at maximum likelihood estimates, and the dashed red line marks half the predicted *wt/wt* HTT level. The shaded "sublinear region" highlights HTT levels below the half threshold. Panels show *Baseline Model*, *Extended Model*, *Extended Model with Nonlinear Translation*, *Extended Model with Clustering*, *Extended Model with Clustering and Nonlinear Translation*, and variants incorporating clustering without CAG repeat length dependence (non-l-dep.). Models with l-dependent clustering better reproduce the sublinear HTT level in *wt/Q111* mice.

dependent clustering could be a relevant regulatory feature in the context of Huntington's disease, although further experimental validation will be required.

10 Effect of the Hill function on translation dynamics across CAG repeat lengths

In the simulation study of the *Extended Model with Clustering and Nonlinear Translation* (l-dependent) (Fig. 21), as the CAG repeat length increases, the total HTT level follows an overall increasing trend, but there are local minima at which the HTT level is lower, despite a higher CAG repeat length. This dynamic is driven by the opposing trends of global

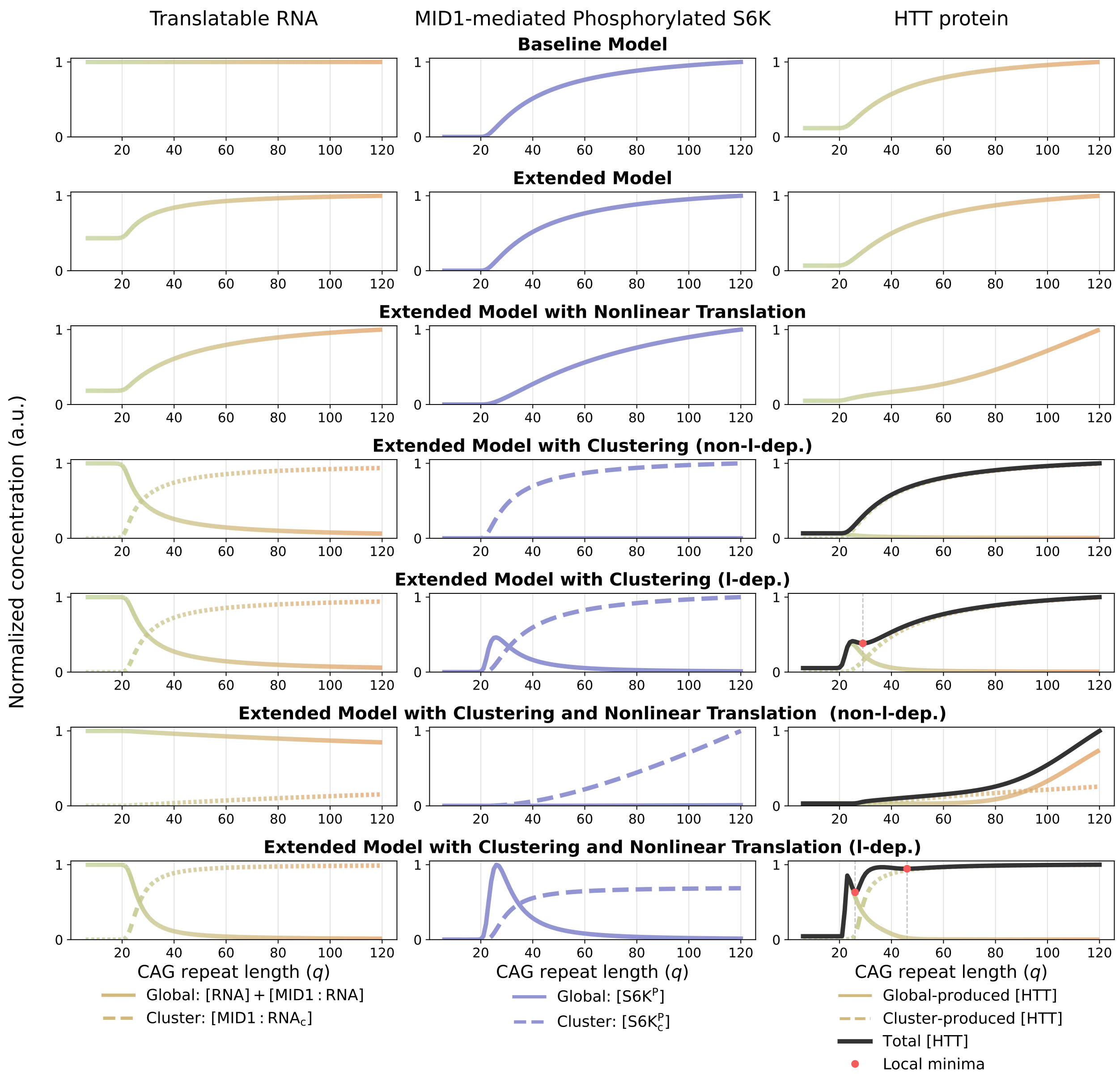

Figure 21: **Simulation studies for all models with varying CAG repeat length.** Steady state simulations for $q_1 = q_2 \in [6, 120]$: left, translatable RNA in global and cluster pools; middle, phosphorylated S6K in both pools; right, HTT levels in each pool and in total with local minima indicated.

and cluster MID1-mediated $\mathrm{S6K^P}$ levels: as q increases, $[\mathrm{S6K^P}]$ declines and $[\mathrm{S6K^P_c}]$ increases, with the Hill-type translation dependence converting these changes into compartment-specific translation rates.

To understand the HTT level dynamics described above, we examined the Hill function for both the global and cluster compartments at selected CAG repeat lengths: low translation

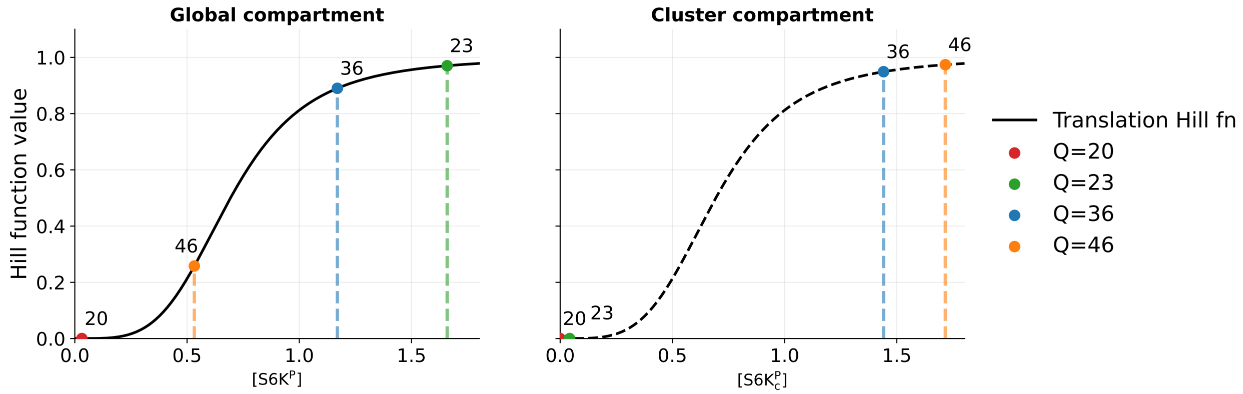

Figure 22: **Effect of the Hill function on translation dynamics across CAG repeat lengths.** Hill function output for global and cluster compartments at $q = 20$ (low translation in both), $q = 23$ (sharp post-threshold increase), $q = 36$ (peak after first drop), and $q = 46$ (second local minimum).

in both compartments ($q = 20$), peak translation in the global compartment ($q = 23$), peak translation after first drop ($q = 36$) and second local minimum of translation ($q = 46$) (Fig. 22).

Before $q = 20$, the threshold of the l function causes the plateau regions for all species, i.e., the steady state of all species does not change when CAG repeats increase. At $q = 20$, there is minimal binding of MID1 to RNA in the global compartment. Thus, there are low amounts of MID1:RNA that could phosphorylate S6K in both compartments, leading to low values of the Hill function. From $q = 20$ to $q = 23$, both compartments show an increase in phosphorylated S6K once the threshold of the l function is crossed, but the effect is asymmetric. In the global compartment, $[\mathrm{S6K^P}]$ rises steeply through the nonlinear part of the Hill curve, producing a strong boost. In contrast, the cluster compartment changes only modestly. This is because (i) clustering can only occur after MID1 has already bound RNA, and (ii) the clustering rate ($c_8 \cdot l$) is generally slower than the binding rate ($c_1 \cdot l$), limiting the overall impact. This change drives the sharp increase in total translation after $q = 20$. Between $q = 36$ and $q = 46$, the cluster compartment is already in the saturation regime of its Hill curve, so translation increases only modestly. In contrast, the global compartment moves further down the declining $[\mathrm{S6K^P}]$ regime, dropping below the midpoint of its Hill curve and causing a steep translation loss. This interplay explains the secondary dip in total translation described in the main text.

In the *Extended Model with Clustering* (l-dependent), the simulation results resemble those of the *Extended Model with Clustering and Nonlinear Translation* (l-dependent) (Fig. 21), with one key difference: the former lacks the second local minimum. This distinction arises because translation in the clustering-only model is governed by a linear rate function, which

produces smoother dynamics. By contrast, the Hill-type function in the nonlinear translation model introduces threshold and saturation effects, allowing additional inflection points and thus the appearance of a second local minimum.

Overall, this analysis confirms that the shape of the Hill curve drives distinct phases of translation dynamics across CAG repeat lengths, resulting in the non-monotonic trends of the two models mentioned above. At the same time, these patterns may partly reflect artifacts of the current model formulation, and additional experimental data will be essential to confirm or refute them.

11 Complete Parameter Table

We summarize all parameters used in each model in our work in the Table 3. As the data were obtained from five independent Western blot gels, gel-specific variability-arising from differences in experimental conditions, sample handling, or imaging sensitivity-must be considered. Thus, in addition to the kinetic parameters and initial conditions, our framework includes additional five independent scaling factors ($s_1, \ldots, s_5$), each corresponding to a distinct western plot gel on which subsets of experiments were measured. Experiments run on the same gel share a scaling factor, as their band intensities are directly comparable, while experiments from different gels require separate scaling factors to account for inter-gel variation. For example, Q17 and Q49 originate from the same gel and thus share one scaling factor, whereas Q20, Q20 si8, and Q20 si9 were measured together on another gel and share a different scaling factor.

Similarly, to account for variability across the five western plot gels, we assume five distinct noise parameters ($\sigma_1, \ldots, \sigma_5$), one for each gel, so that every dataset is modeled with its own measurement noise distribution. This approach avoids the bias that would result from imposing a single global noise level across all experiments. The trade-off is increased model complexity, which can aggravate identifiability or overfitting, and in principle, too many noise parameters could distort the likelihood landscape if residuals reached zero (since $\sigma \to 0$ would make it diverge), making optimization towards the optimal point difficult. In our case, however, multiple replicates that are different from each other prevent this situation, as the residuals would never be zero.

To assess whether the assumption of gel-specific noise parameters is reasonable, we examined residual diagnostics across all models (Fig. 23). The histograms and Q–Q plots show that residuals are approximately centered, with no major systematic biases. While the simpler models leave heavier tails and visible structure, the extended models with clustering and nonlinear translation yield more symmetric, near-normal residuals with reduced patterns. This supports the adequacy of the adopted noise model and suggests that our inference

Table 3: **Parameters.** Parameter, their associated reaction or definition, bounds, and prior distribution used for optimization. All parameters are fitted. *Units:* UC = unit of concentration; UT = unit of time; "–" = dimensionless.

Reaction or Definition	Name	Lower Bound	Upper Bound	Prior	Unit
Binding	c_1	1E−6	1E8	log-uniform	$UC^{-1} \cdot UT^{-1}$
Unbinding	c_{-1}	1E−6	1E8	log-uniform	UT^{-1}
Phosphorylation	c_2	1E−6	1E8	log-uniform	$UC^{-1} \cdot UT^{-1}$
Unphosphorylation	c_{-2}	1E−6	1E8	log-uniform	$UC \cdot UT^{-1}$
	K_{-2}	1E−14	1E8	log-uniform	UC
Translation	c_3	1E−6	1E8	log-uniform	UT^{-1}
	ξ_3	1E0	1E2	log-uniform	–
	n_3	1	4	uniform	–
	K_3	1E−6	1E8	log-uniform	UC
HTT Degradation	c_4	1E−6	1E8	log-uniform	UT^{-1}
Transcription	c_5	1E−6	1E8	log-uniform	$UC \cdot UT^{-1}$
RNA Degradation	c_6	1E−6	1E8	log-uniform	UT^{-1}
	ξ_6	1E0	1E5	log-uniform	–
MID1:RNA Degradation	c_7	1E−6	1E8	log-uniform	UT^{-1}
Clustering	c_8	1E−6	1E8	log-uniform	UT^{-1}
Declustering	c_{-8}	1E−6	1E8	log-uniform	UT^{-1}
wt CAG repeat length	Q_{wt}	7	36	uniform	–
$[RNA]_0$	rna_0	1E−6	1E8	log-uniform	UC
$[S6K]_0$	$s6k_0$	1E−6	1E8	log-uniform	UC
$[S6K_c]_0/[S6K]_0$	$\xi_{[S6K_c]_0}$	1E−3	1E0	log-uniform	–
siRNA si-8 effect	$si8_{effect}$	−10	0	uniform	–
siRNA si-9 effect	$si9_{effect}$	−10	0	uniform	–
scaling factor 1	s_1	1E−6	1E8	uniform	–
scaling factor 2	s_2	1E−6	1E8	uniform	–
scaling factor 3	s_3	1E−6	1E8	uniform	–
scaling factor 4	s_4	1E−6	1E8	uniform	–
scaling factor 5	s_5	1E−6	1E8	uniform	–
noise parameter	σ_1	0	∞	uniform	–
noise parameter	σ_2	0	∞	uniform	–
noise parameter	σ_3	0	∞	uniform	–
noise parameter	σ_4	0	∞	uniform	–
noise parameter	σ_5	0	∞	uniform	–

framework can capture the main sources of variability in the data.

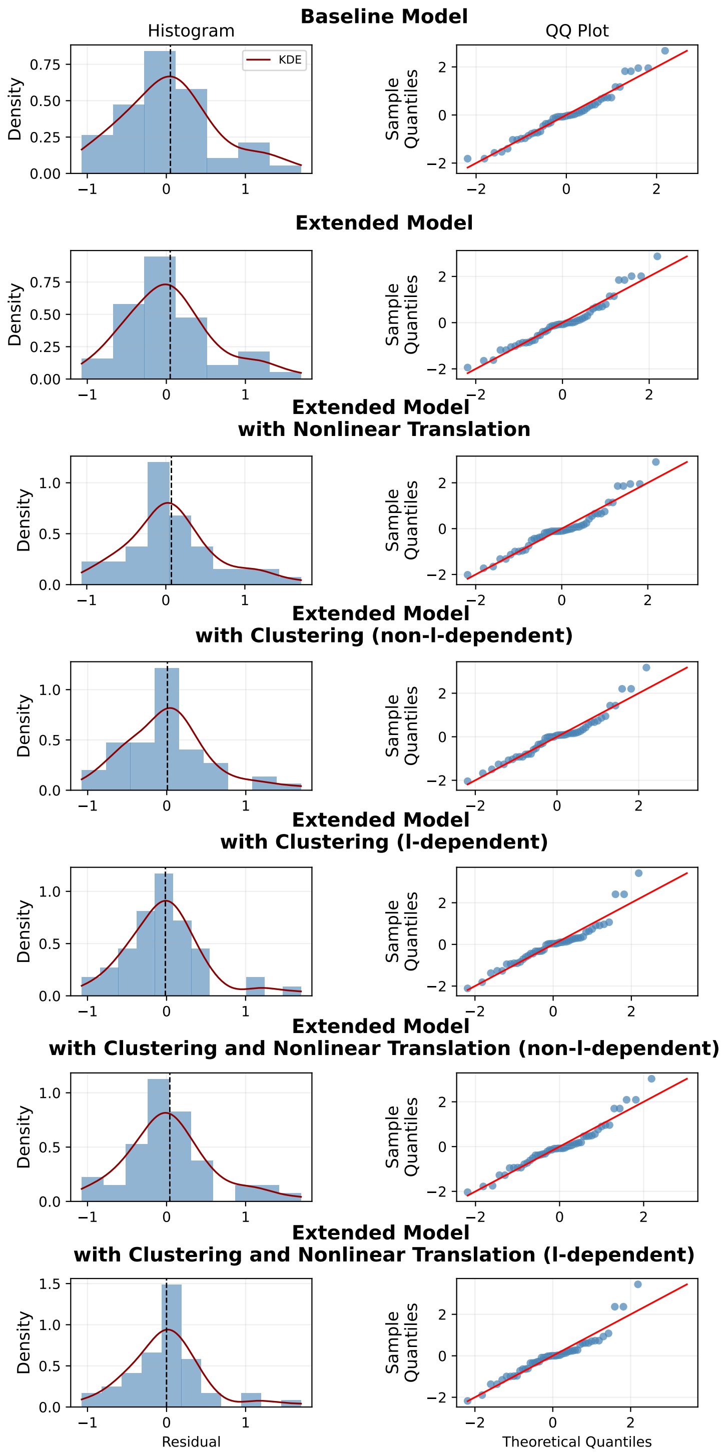

Figure 23: **Residual diagnostics for all models.** Histograms with kernel density estimates (left) and Q–Q plots (middle) are shown for each model. Improvements in normality and reduction of systematic residual patterns are most apparent in the extended models with clustering and nonlinear translation.

References

[1] S. Krauß, N. Griesche, E. Jastrzebska, C. Chen, D. Rutschow, C. Achmüller, S. Dorn, S. M. Boesch, M. Lalowski, E. Wanker, and R. Schneider. Translation of HTT mRNA with expanded CAG repeats is regulated by the MID1-PP2A protein complex. *Nature Communications*, 4:1511, 2013.

[2] T. S. Mahendran, G. M. Wadsworth, A. Singh, R. Gupta, and P. R. Banerjee. Homotypic RNA clustering accompanies a liquid-to-solid transition inside the core of multicomponent biomolecular condensates. *Nature Chemistry*, pages 1–11, 2025.
